# Coal Mining Activities Driving the Changes in Microbial Community and Hydrochemical Characteristics of Underground Mine Water

**DOI:** 10.3390/ijerph192013359

**Published:** 2022-10-16

**Authors:** Li Zhang, Zhimin Xu, Yajun Sun, Yating Gao, Lulu Zhu

**Affiliations:** 1 School of Resources and Geosciences, China University of Mining and Technology, Xuzhou 221116, China; 2 Fundamental Research Laboratory for Mine Water Hazards Prevention and Controlling Technology, Xuzhou 221006, China

**Keywords:** coal mining impacts, hydrochemical characteristics, microbial communities, zone-specific patterns, biogeochemical process

## Abstract

Coal mining can cause groundwater pollution, and microorganism may reflect/affect its hydrochemical characteristics, yet little is known about the microorganism’s distribution characteristics and its influence on the formation and evolution of mine water quality in underground coal mines. Here, we investigated the hydrochemical characteristics and microbial communities of six typical zones in a typical North China coalfield. The results showed that hydrochemical compositions and microbial communities of the water samples displayed apparent zone-specific patterns. The microbial community diversity of the six zones followed the order of surface waters > coal roadways > water sumps ≈ rock roadways ≈ goafs > groundwater aquifers. The microbial communities corresponded to the redox sensitive indices’ levels. Coal roadways and goafs were the critical zones of groundwater pollution prevention and control. During tunneling in the panel, pyrite was oxidized by sulfur-oxidizing bacteria leading to SO_4_^2−^ increase. With the closure of the panel and formation of the goaf, SO_4_^2−^ increased rapidly for a short period. However, with the time since goaf closure, sulfate-reducing bacteria (e.g., *c_Thermodesulfovibrionia*, *Desulfobacterium_catecholicum*, etc.) proportion increased significantly, leading to SO_4_^2−^ concentration’s decrease by 42% over 12 years, indicating the long-term closed goafs had a certain self-purification ability. These findings would benefit mine water pollution prevention and control by district.

## 1. Introduction

Coal mining usually produces a large amount of mine water and may cause groundwater pollution [1,2,3,4,5]. Clarifying the hydrochemical characteristics of mine water and its influencing factors is the theoretical basis of groundwater pollution prevention and control in coal mining areas. In groundwater systems, microbial communities, as the main driving factors of the biogeochemical cycle, are the important undertakers of material cycle, energy conversion, and information transfer [6]. The specificity of microbial community composition could reflect and affect the environmental physicochemical characteristics of groundwater [7,8,9]. The reported studies suggested that microbial communities play an important role in environmental changes in mining areas and focused on the formation of acid mine drainage (AMD) and the effects of mine drainage on the microbial community structures and hydrochemical characteristics of surface ecosystems, such as soils and rivers [10,11,12,13,14,15]. Microbial activities mainly affect the fate of redox sensitive compounds in mine water, including SO_4_^2−^, NO_3_^−^, NO_2_^−^, NH_4_^+^, Fe, Mn, etc. [16,17]. During coal mining, some sulfide minerals in coal were oxidized and hydrolyzed with the participation of various microorganisms, leading to the generation of sulfuric acid and iron hydroxide, and even the formation of AMD (pH < 6), with high concentrations of SO_4_^2−^ and heavy metal [18]. Zhang et al. (2019) [14] reported that the diversities of bacterial communities significantly increased along the river, with decreasing contamination when AMD flooded into Hengshi River, and the relative abundances of the bacteria that functioned in metal reduction decreased along the AMD gradient. Moreover, microbial communities in the rivers receiving alkaline mine drainage (AlkMD) were fundamentally different from AMD [15]. Jin et al. (2022) [15] found that AlkMD had a positive impact on alpha diversity of the impacted river bacterial communities, but also led to the loss of some predicted microbial metabolic. However, in underground coal mines, the microbial community and its response or effect on hydrochemical characteristic have remained largely unexplored.

During underground coal mining, for production safety, the groundwater from the coal seam roof and floor is continuously drained, which enhances the hydraulic connection of each aquifer. Meanwhile, oxygen is introduced into the mine by ventilation, which increases the dissolved oxygen (DO) concentration of the mine water. These mining disturbances increase the microbial biomass and diversity of the mine water [19]. Ben Maamar et al. (2015) [20] reported that the mixing of groundwater in different ages strongly affected the community structure and favored iron oxidation. A large number of the fresh coal and gangue fragments formed by coal mining would increase the specific surface area of the water–coal/rock reaction, possibly leading to the dissolution of the organic compounds in coal (e.g., aliphatic and aromatic hydrocarbons) into the mine water, as well as the promoted growth of these organic compound degradation bacteria. Gao (2019) [21] and Zhang (2019) [22] screened many polycyclic aromatic hydrocarbon (PAH) dominant degradation bacteria from mine sludge using phenanthrene and catechol as substrates. At the same time, some microorganisms that are initially attached to coal solids could migrate into mine water. Coal mining may also affect subsurface microbial populations by introducing new species (e.g., drug-resistant microbes) from the mine infrastructure, mining equipment, and personnel. Furthermore, the mining disturbances and provenance characteristics of different zones in the coal mine (e.g., rock roadway, coal roadway, goaf, water sump, etc.) were different. In particular, coal mining is mainly carried out on the tunneling panel of the coal roadway, which contains a large amount of coal. Thus, in the coal roadway, more organic matter and sulfide minerals from coal may provide more nutrients and electron donors for microorganism metabolic activities. After cessation of mining, a closed wall is be built to close the goaf. Then, in the goaf, the water level increases gradually, and the oxygen concentrations decreases gradually, resulting in a long-term and specific water–coal/rock reaction. Therefore, the mine water in different zones may form some unique microbial communities, which might, in turn, affect the formation and evolution of mine water quality. However, few studies have been reported on this subject.

Here, in each of the different zones (i.e., groundwater aquifer, rock roadway, coal roadway, goaf, water sump, surface water), water samples were collected for microbial community and hydrochemical analysis. Multivariate statistical methods were used to investigate the relationships between microbial communities and spatial hydrochemical variables. The main objectives of this study were to: (1) describe the effects of coal mining on microbial communities and hydrochemical characteristics of mine water in a typical North China coalfield; (2) illustrate the relationships between microbial communities and hydrochemical characteristics; (3) elucidate the variation mechanism of microbial communities in different zones and their response or effect on hydrochemical components. Such knowledge may be beneficial for clarifying the formation and evolution mechanisms of the special mine water quality in different zones and developing microbial treatment technologies for mine water.

## 2. Materials and Methods

### 2.1. Study Sites and Sample Collection

**Figure 1:**
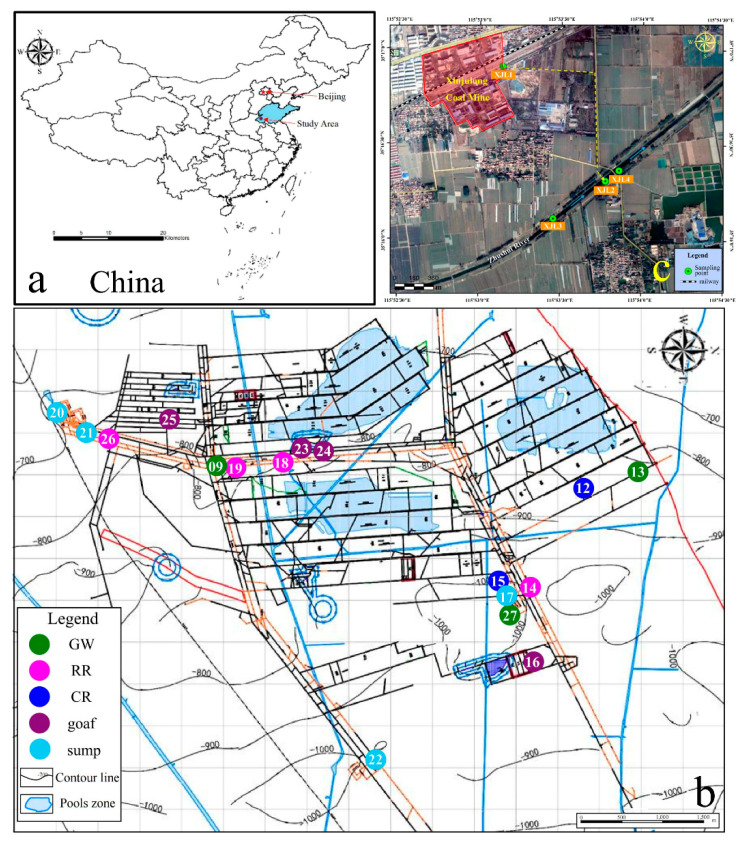
Locations of sampling sites in Xinjulong coal mine and mine-water-discharged Zhushui River in Shandong Province, China. Xinjulong coal mine is located in Shandong Province, China (**a**). Sampling sites included 17 underground sites (**b**) and 4 surface water sites (**c**). Note: The number in image b (XJL is omitted) represents the sample point name, and detailed sampling place is shown in Table S1. GW, RR, CR, goaf, sump, and SW refer to groundwater aquifers (XJL9, XJL13, XJL27), rock roadways (XJL14, XJL18, XJL19, XJL26), coal roadways (XJL12, XJL15), goafs (XJL16, XJL23, XJL24, XJL25), water sumps (XJL17, XJL20, XJL21, XJL22), and surface waters (XJL1, XJL2, XJL3, XJL4), respectively. The blue and yellow arrows refer to the flow direction of the river and the mine drainage, respectively.

**Figure 2:**
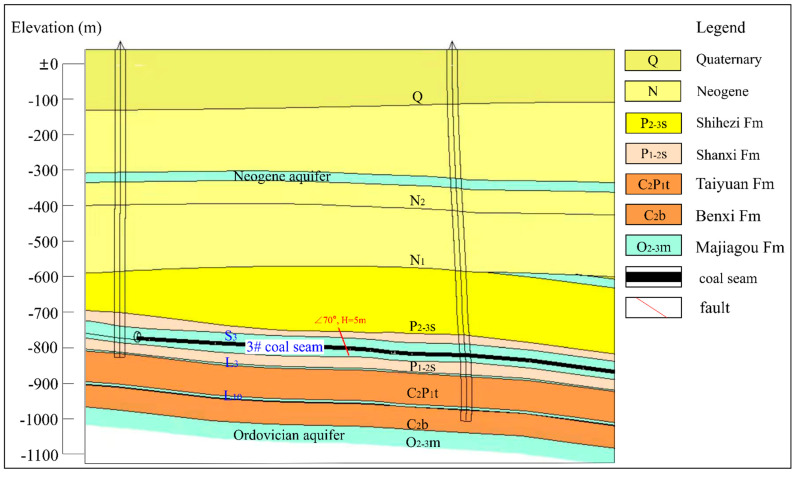
Stratigraphic profile of 3# coal in Xinjulong coal mine.

This study was conducted at Xinjulong coal mine, located in North China (Longgu Town, Juye County, and Shandong Province) (Figure 1). This area belongs to the alluvial proluvial plain of the Yellow River, where the surface water system is relatively developed, including Zhushui River, which receives the mine drainage. Xinjulong coal mine has been in operation since 2009, with a production capacity of 6–7.5 million tons per year. This mine is a fully concealed North China Carboniferous–Permian coalfield. Its main coal-bearing strata are Shanxi Formation and Taiyuan Formation, and the 3# coal of Shanxi Formation is the main mining seam, with a depth of 800–1300 m. In recent years, the mine water inflow was 1109–1210 m^3^/h, and the main mine water was originated from the roof and floor aquifers of 3# coal, including Shanxi Formation sandstone (S_3_) and Taiyuan Formation limestone (L_3_) aquifers (Figure 2). The groundwater circulation was slow, and the hydraulic connection between aquifers was poor.

The mine water, coming from groundwater aquifers, flowed through rock roadway or coal roadway (including panel) or goaf, in turn, and then was collected to the water sump, followed by being discharged to the Zhushui River. In order to research the whole process of mine water formation, collection, and discharge, the water samples in relevant areas were sampled, and the detail sampling points design and their different environments description were shown in Supplementary Material. Thus, 21 water samples were collected from different groundwater aquifers (GW: XJL9, XJL13, XJL27), rock roadways (RR: XJL14, XJL18, XJL19, XJL26), coal roadways (CR: XJL12, XJL15), goafs (goaf: XJL16, XJL23, XJL24, XJL25), water sumps (sump: XJL17, XJL20, XJL21, XJL22), and surface waters (SW: XJL1, XJL2, XJL3, XJL4) of Xinjulong coal mine in May 2021 for hydrochemical and microbial analysis (Figure 1b,c, Supplementary Figure S1 and Supplementary Table S1). Samples for hydrochemical analysis were collected and stored in 500 mL and 2 L polyethylene bottles. These water samples were filtered through 0.45 μm pre-sterilized PES membrane (Tianjin Jinteng Experimental Equipment Co., Ltd., Tianjin, China) in the field. The water samples in 500 mL polyethylene bottles were used to analyze cations, and guaranteed reagent HNO_3_ was added to adjust the pH below 2. Samples for microbial analysis were collected in 5 L sterile polyethylene bottles. In order to collect biomass, these water samples were filtered through 0.22 μm pre-sterilized PES membrane (Tianjin Jinteng Experimental Equipment Co., Ltd.) immediately or stored in refrigerator at 4 °C within 48 h before filtration. The biomass was transported to the molecular biology laboratory immediately under dry ice storage and stored at −80 °C until DNA extraction.

### 2.2. Hydrochemical Characterization

Hydrochemical characterization was measured by both in situ test and laboratory analysis. Parameters of temperature, pH, oxidation-reduction potential (ORP), DO, and turbidity were tested in situ by HI9829 multi-parameter water quality analyzer (Hanna Instruments, Limena, Italy), with a precision of ±0.15 °C for temperature, ±0.02 for pH, ±1.0 mv for ORP, ±0.10 mg/L for DO, and ±0.3 FNU for turbidity. This water quality analyzer was calibrated and verified in situ before the measurements. Free CO_2_ and HCO_3_^−^/CO_3_^2−^ were measured in laboratory within 48 h after sampling using alkali titration and acid titration, respectively (automatic potentiometric titrator, Metrohm, Switzerland). Na^+^, K^+^, Ca^2+^, and Mg^2+^ were detected by inductively coupled plasma optical emission spectrometer (ICP-OES, Spectro Analytical Instruments, Germany). NO_3_^−^, NO_2_^−^, NH_4_^+^, Fe^2+^, Fe^3+^, and dissolvable silica were analyzed by spectrophotometer (UV-1900, Shimadzu, Japan). SO_4_^2−^ and Cl^−^ were detected by barium sulfate gravimetric and silver nitrate titration methods, respectively. Total dissolved solids (TDS) were measured by gravimetric method. Chemical oxygen demand (COD) was analyzed by potassium permanganate index method [23]. The precision of these analytical methods is shown in Supplementary Table S1. The electroneutrality error values of all samples were less than 5%, indicating that the data were credible. OriginPro 2022 and AqQA soft were used to construct the box plots and the piper and durov diagrams, respectively.

### 2.3. DNA Extraction and 16S rRNA Gene Sequencing

Microbial community genomic DNA was extracted using the FastDNA SPIN kit for soil (MP Biomedical, Santa Ana, CA, USA), according to manufacturer’s instructions. Then, the quality of the exacted DNA was checked on 1% agarose gel, and the concentration and purity of total DNA were measured by a NanoDrop 2000 UV–Vis spectrophotometer (Thermo Scientific, Wilmington, DE, USA). The hypervariable region V3–V4 of the bacterial 16S rRNA gene were amplified with primer pairs 338F (5′-ACTCCTACGGGAGGCAGCAG-3′) and 806R(5′-GGACTACHVGGGTWTCTAAT-3′) by an ABI GeneAmp^®^ 9700 PCR thermocycler (ABI, Santa Ana, CA, USA). PCR amplification was performed using *TransStart* Fastpfu DNA polymerase (20 μL) containing 5 × FastPfu buffer (4 μL), 2.5 mM dNTPs (2 μL), 5 μM forward primer (0.8 μL), 5 μM reverse primer (0.8 μL), FastPfu polymerase (0.4 μL), template DNA (10 ng), and finally, ddH_2_O up to 20 μL. The PCR amplification of 16S rRNA gene was performed as follows: initial denaturation at 95 °C for 3 min, followed by 27 cycles of denaturing at 95 °C for 30 s, annealing at 55 °C for 30 s, extension at 72 °C for 45 s, single extension at 72 °C for 10 min, and ending at 4 °C. PCR reactions were performed in triplicate. The PCR products were extracted from 2% agarose gel and purified using the AxyPrep DNA gel extraction kit (Axygen Biosciences, Union City, CA, USA), according to manufacturer’s instructions, and quantified using Quantus™ fluorometer (Promega, Madison, WI, USA). Purified amplicons were pooled in equimolar and paired-end sequenced on an Illumina MiSeq PE300 platform (Illumina, San Diego, CA, USA), according to the standard protocols by Majorbio Bio-Pharm Technology Co., Ltd. (Shanghai, China). The raw reads were submitted into the NCBI Sequence Read Archive database (accession number: PRJNA818133).

### 2.4. Processing and Statistical Analyses of Sequence Data

The original 16S rRNA gene sequencing reads were demultiplexed, quality-filtered by fastp 0.20.0 [24], and merged by Flash 1.2.11 [25]. Operational taxonomic units (OTUs) with 97% similarity cutoff were clustered using Uparse 7.0.1090 [26], and chimeric sequences were identified and removed. The taxonomy of each OTU representative sequence was analyzed by RDP classifier 2.11 from the Silva v138 16S rRNA database, with confidence threshold of 0.7 [27].

Alpha diversity indexes, including Sobs, ACE, Chao1, Shannon, and Simpson, were calculated on Mothur 1.30.2. Before analyzing alpha diversity, rarefaction curves were obtained by R language (Supplementary Figure S2), showing that the observed OTU numbers were close to saturation when the number of reads in each sample exceeded 20,000. The species Venn diagram and the relative abundance of dominant lineages, and community heatmap (phylum/genus level) for water samples were displayed by R language 3.3.1. Kruskal–Wallis H test was used to test the significant difference between different groups. Beta diversity analysis was also conducted on R language 3.3.1. Based on unweighted UniFrac full-tree dissimilarity, principal co-ordinates analysis (PCoA) was used to simplify the microbial community of water samples in multidimensional space to low-dimensional space for visualization of microbial community’s dissimilarity. Analysis of similarities (ANOSIM) was used to test whether the difference between six groups was significantly greater than the difference within the group using unweighted UniFrac distance algorithm. Redundancy analysis (RDA) was used to analyze the relationships between microbial communities and environmental factors, so as to explore the influence of environmental factors on microbial community structures of water samples. Environmental factors and the OTU abundance dataset were two input datasets, and calculation and visualization of RDA were performed by vegan package of R language. Correlation heatmap analysis was to calculate the spearman correlation coefficient between environmental factors and the top 50 species in total abundance and visually display the obtained numerical matrix through the heatmap on pheatmap package of R language. Biotic interactions were visualized by co-occurrence networks representing these interactions in different zones of Xinjulong coal mine. The co-occurrence networks were visualized using the interactive platform Gephi 0.9.2 [28]. The 100 most abundant OTUs from water samples were selected to construct the cooccurrence networks. Each connecting line indicated a strong (|r| > 0.6) and significant (*p* < 0.05) spearman’s correlation [29]. Functional annotation of prokaryotic taxa (FAPROTAX) prediction was conducted to predict the function of microorganisms. Based on the functional annotation of prokaryotic taxa (database), FAPROTAX can predict functional groups, metabolic phenotypes, or ecological functions [30]. FAPROTAX prediction focuses on the biogeochemical cycle of S, N, H, and C. The FAPROTAX database and associated software are freely available at www.zoology.ubc.ca/louca/FAPROTAX (accessed on 2 October 2021).

## 3. Results and Discussion

### 3.1. Spatial Changes in Hydrochemical Characteristic

**Figure 3:**
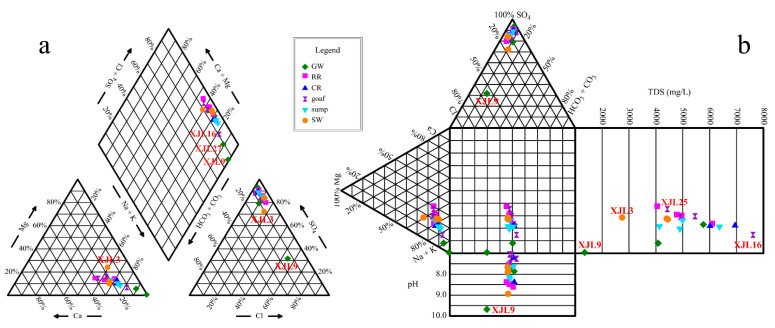
Piper (**a**) and durov (**b**) diagrams of the water samples. The points with same colors and shapes represent water samples from the same zones. Note: GW, RR, CR, goaf, sump, and SW refer to groundwater aquifers, rock roadways, coal roadways, goafs, water sumps, and surface waters, respectively.

**Figure 4:**
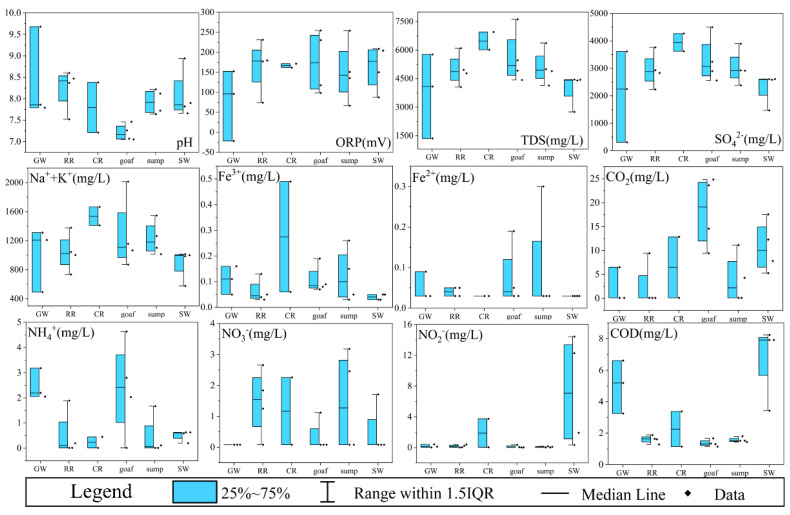
Half-box-and-whisker diagrams of the major hydrochemical components in the six zones. Note: GW, RR, CR, goaf, sump, and SW refer to groundwater aquifers, rock roadways, coal roadways, goafs, water sumps, and surface waters, respectively.

The hydrochemical characteristics of the groundwater and mine water in different zones are shown in Figure 3 and Figure 4, Supplemental Figure S3 and Table S1. Piper and durov diagrams indicated that S_3_ sandstone and L_3_ limestone groundwater samples were clustered, which were the main recharge source of mine water. The chemical types of these two groundwater were both SO_4_-Na type. The water samples of rock roadways, coal roadways, goafs, and water sumps were also clustered, and their hydrochemical types were consistent with those of their recharge water source (SO_4_-Na type). However, the Ordovician limestone groundwater sample (XJL9) was located far from all other samples. Additionally, its hydrochemical type was the Cl·SO_4_-Na type, which indicated there may be a unique biochemical reaction. Among the four surface waters, the mine drainage outlet (XJL1), the intersection of mine drainage and Zhushui River (XJL2), and the downstream (XJL4) of Zhushui River were relatively clustered, while the upstream of Zhushui River (XJL3) was far away from them, suggesting that mine drainage had a significant impact on the chemical composition of the receptor river water.

Hydrochemical components of water samples have been plotted as half-box-and whisker diagrams, based on their locations in different zones, to compare hydrochemical variation among these zones (Figure 4 and Supplemental Figure S3). Based on the median value, the pH values of goafs were the lowest (7.1–7.5), followed by those of coal roadways, groundwater aquifers, surface waters, and water sumps, and the pH values of the rock roadways were the highest (7.5–8.6). The calcium or limestone of coal seam floor and concrete possibly increased the alkalinity of the rock roadway (Figure 4). The TDS of mine water ranged from 4.14 to 7.61 g/L, and the TDS of coal roadways was obviously higher than that of other zones. The most contributions to TDS were SO_4_^2−^ and Na^+^+K^+^, in which the SO_4_^2−^ (3.6–4.3 g/L) and Na^+^+K^+^ (1.4–1.7 g/L) concentrations of the coal roadways were the highest, followed by the SO_4_^2−^ (2.6–4.5 g/L) and Na^+^+K^+^ (0.9–2.0 g/L) of the goafs. Combined with higher Fe^3+^ (ND–0.5 mg/L) and Fe^2+^ (ND–0.2 mg/L) in the coal roadway and goafs, it was inferred that pyrite in the coal could be oxidized to generate a large amount of sulfuric acid and iron hydroxide (Equations (S1)–(S3)). Compared with coal roadways, the SO_4_^2−^ concentration (4.5 g/L) of goaf closure in 2021 (newly closure, XJL16) was higher, and the pH value (7.3) was lower, but the SO_4_^2−^ concentration decreased gradually with the time since goaf closure (2.6 g/L of goaf closure in 2009, XJL25). This interesting phenomenon is discussed in detail in Section 3.4.

The concentrations of NH_4_^+^, NO_2_^−^, and NO_3_^−^, which were closely related to the redox conditions in the environment, were significantly different between the sampling zones (Figure 4). In particular, the NH_4_^+^ concentrations of groundwater aquifers were significantly higher than those of rock roadways and coal roadways, and NO_2_^−^ and NO_3_^−^ were both negatively correlated with NH_4_^+^ (Figure 6a). When groundwater inflowed into rock roadways and coal roadways, nitrification occurred with the increase of oxygen, and then NH_4_^+^ was oxidized to NO_2_^−^ and NO_3_^−^. Additionally, in the goafs, the NH_4_^+^ concentration was generally higher than the NO_2_^−^ and NO_3_^−^ concentrations, which may be related to the closure of the panel and the gradual consumption of oxygen. The COD values were the highest in surface waters, followed by groundwater aquifers and coal roadways, and they were lowest in water sumps, goafs, and rock roadways. In a word, these results highlighted that the hydrochemical environment of different zones water had obvious regional characteristics.

### 3.2. Overall Microbial Diversity and Taxonomic Composition Variation across Different Zones

A total of 1,259,517 valid sequence reads were obtained from 21 water samples after the removal of low-quality and chimeric sequences (Supplemental Table S2). In order to ensure comparability between the different samples, the samples were flattened, according to the minimum number of sequences. All sequences had been assigned to 12,768 valid OTUs, with a 97% similarity. The near-saturated rarefication shown in Supplemental Figure S2 indicated sufficient sequencing depth for the microbial communities of these water samples. The alpha diversity indexes shown in Supplemental Table S2 were employed to interpret microbial community richness and diversity of water samples. The results showed that the microbial community richness of the six zones was ranked as follows: coal roadways > water sumps ≈ surface waters > rock roadways > goafs > groundwater aquifers. The microbial community diversity of the six zones followed the order of surface waters > coal roadways > water sumps ≈ rock roadways ≈ goafs > groundwater aquifers. In addition, as shown in the Venn diagram (Supplemental Figure S4), compared with the underground water samples, the surface waters contained 96 unique species, suggesting that the underground microbial community was quite different from that of the surface ecosystems. Among the underground mine water in the five zones, the microbial species detected in the water sumps were detected the most, followed by the coal roadways and goafs, which also contained the most unique species, and the species detected in the groundwater aquifers were the least. This result also indicated that coal mining possibly had a great impact on the microbial community of mine water, especially in coal roadways and goafs.

**Figure 5:**
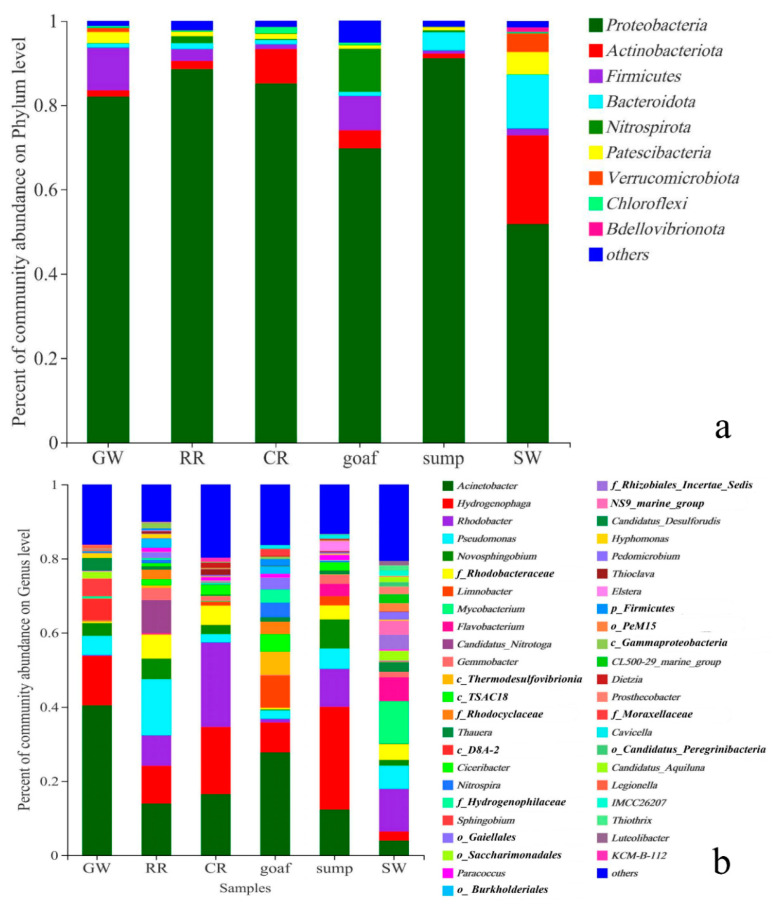
Relative abundance of the dominant lineages on phylum (**a**) and genus (**b**) levels for different zones water samples. The relative abundance of microbial phyla/genera >1% was plotted, and the remaining microorganisms were classified as others. Note: GW, RR, CR, goaf, sump, and SW refer to groundwater aquifers, rock roadways, coal roadways, goafs, water sumps, and surface waters, respectively.

Within 21 water samples, a total of 52 phyla bacteria were detected, and the top nine most abundant phyla accounted for up to 99% of the total microbial community (Figure 5a and Supplemental Table S3). Taxonomic analysis revealed that *Proteobacteria* (51.82–91.16%) was the most abundant phylum. This finding was consistent with other reports that also stated that *Proteobacteria* predominated in mining areas, aquifers, and rivers [31,32]. Additionally, the other abundant phyla include *Actinobacteria* (1.08–20.96%), *Firmicutes* (0.73–10.15%), *Bacteroidota* (0.95–12.80%), *Nitrospirota* (0.01–10.24%), *Patescibacteria* (0.71–5.32%), and *Chloroflexi* (0.12–1.56%). The Kruskal–Wallis H test results showed that the abundance of *Nitrospirota* was significantly different, in terms of the six different zones (*p* < 0.05, Supplemental Figure S6a). The relative abundances of *Nitrospirota* in the goafs and rock roadways were higher than other zones. *Actinobacteria* was more predominant in the surface waters and coal roadways, possibly because most of the *Actinobacteria* were aerobic saprophytic bacteria. The relative abundance of *Firmicutes* was higher in the groundwater aquifers and goafs, many of which could adapt well to extreme environments and degrade organic pollutants [33].

On genus level, *Acinetobacter* (3.94–40.46%), *Hydrogenophaga* (2.43–27.71%), *Rhodobacter* (0.23–22.76%), *Pseudomonas* (2.27–15.13%), *Novosphingobium* (0.23–7.85%), and *Limnobacter* (0.13–8.63%) were of predominant abundance (Figure 5b and Supplemental Table S3). The Kruskal–Wallis H test result indicated that the abundances of *Flavobacterium, c_Thermodesulfovibrionia, f_Rhodocyclaceae*, and *Pedomicrobium* were significantly different, in terms of the six different zones (*p* < 0.05, Supplemental Figure S6b). *Flavobacterium* and *Pedomicrobium*, as the aerobic bacteria, accounted for the higher proportion in surface waters and water sumps. However, *c_Thermodesulfovibrionia*, which could reduce SO_4_^2−^, and *f_Rhodocyclaceae*, which could degrade aliphatic and aromatic hydrocarbons [19], were predominant in goafs and rock roadways. *Thauera* and *Limnobacter* could also degrade many aromatic pollutants and were widely distributed in mine water, especially in goafs and water sumps (1.02–8.63%), but they accounted for the lower proportion in groundwater aquifers. *Thioclava* (1.56%) and *Dietzia* (1.39%) were more abundant in coal roadways; the former could accelerate the oxidation of pyrite, while the latter had a good performance in hydrocarbon degradation and good tolerance to high salt and alkali. This finding corresponded to the high sulfur and high organic matter environment of the coal roadways. Anyway, the abundances of *Candidatus_Nitrotoga* and *Nitrospira* were higher in the rock roadways (0.89–8.98%) and goafs (0.08–3.92%), which was speculated to be related to the higher concentration of NH_4_^+^ or NO_3_^−^ and stronger nitrogen conversion in these two zones. *Sphingobium* and *Candidatus desulforudis* were almost exclusively distributed in groundwater aquifers, with abundances of 4.78% and 3.43%, respectively. Meanwhile, aerobic *Mycobacterium* was the dominant species only in surface waters.

### 3.3. Relationships between Microbial Community Structure and Hydrochemical Components

**Figure 6:**
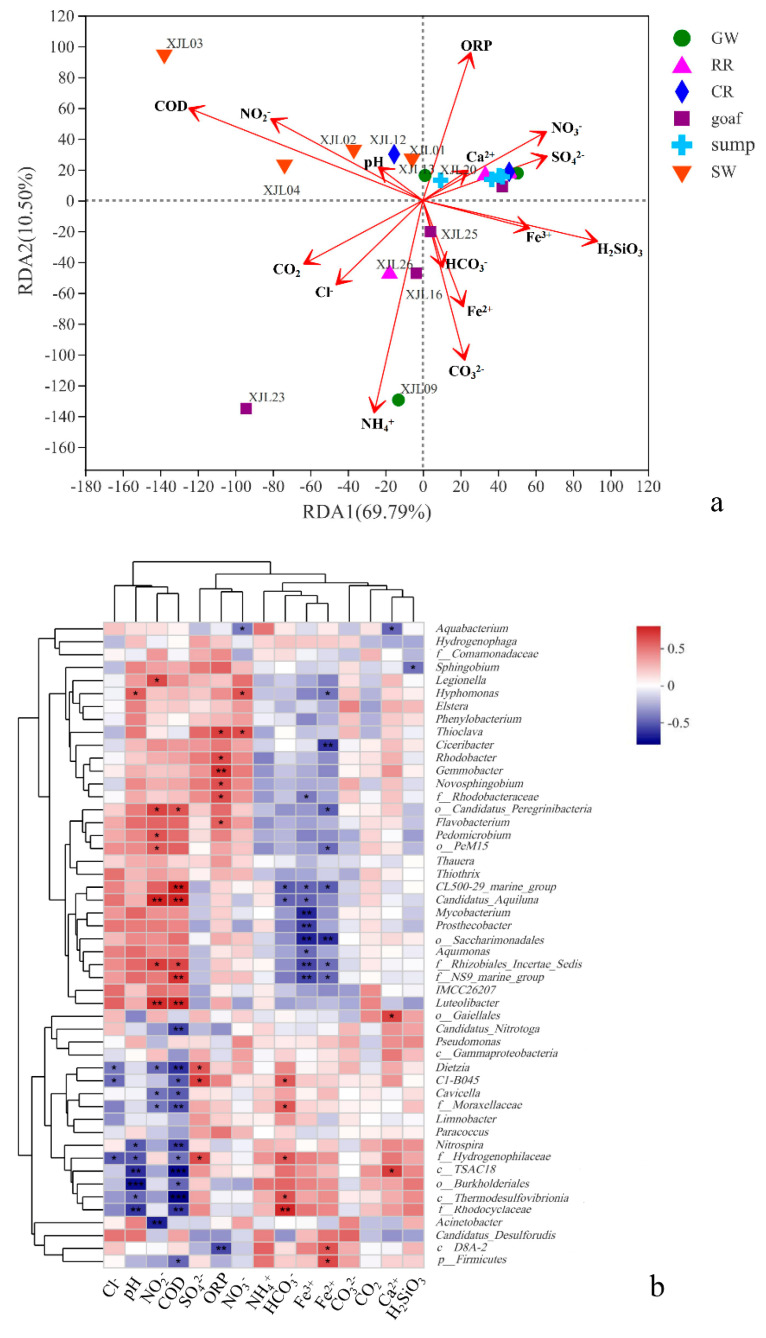

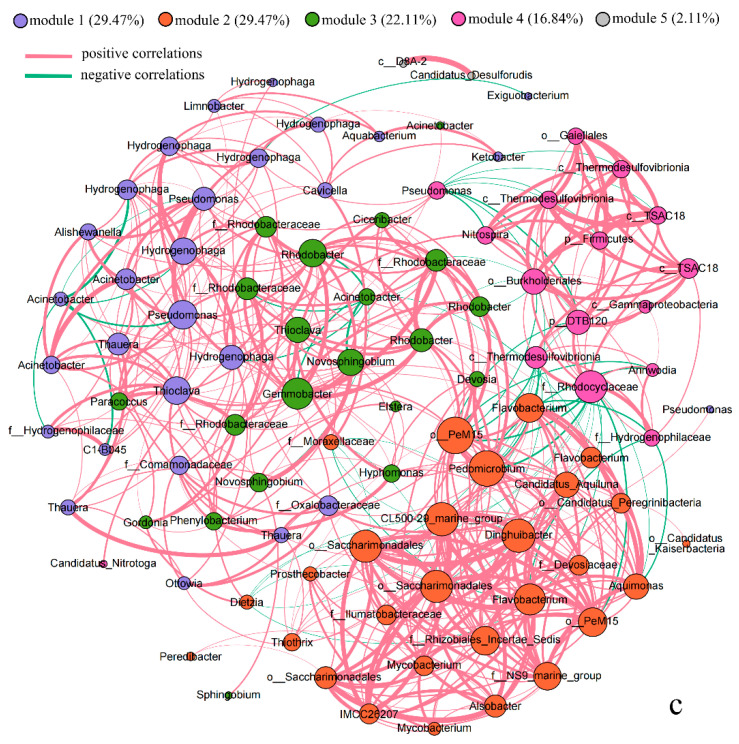
(**a**) RDA of the relations between the selected environmental variables and microbial communities in water samples. The red arrows represent the environmental variables, the length of which can represent the impact degree of environmental factor on species data. The angle between the different environmental factors arrows represents positive (acute angle) or negative (obtuse angle) or no correlation (right angle) between the environmental factors. Points with different colors or shapes represent different water samples, and the distances between points represent the similarity of the two samples. When projecting from the sample point to the environmental factor arrow, the distance from the projection point to the origin represents the relative impact of environmental factor on the sample community distribution. (**b**) Heatmap of Spearman’s rank correlation coefficients between environmental variables and the top 50 microbial phylotypes on genus levels. The correlation coefficients are indicated by hue (* 0.01 < *p* ≤ 0.05, ** 0.001 < *p* ≤ 0.01, *** *p* ≤ 0.001). Note: GW, RR, CR, goaf, sump, and SW refer to groundwater aquifers, rock roadways, coal roadways, goafs, water sumps, and surface waters, respectively. (**c**) Network of co-occurrence bacterial genera, based on correlation analysis (top 100 most abundant OTUs). The size of each node is proportional to the number of connections; the thickness of each connection between two nodes is proportional to the absolute value of Spearman’s correlation coefficients. The connections indicate strong (|r| > 0.6) and significant (*p* < 0.05) Spearman correlations.

The RDA and heatmap were used to explore the relationship between microbial community and environment variables and evaluate the potential impacts on the microbial community. After deleting 5 collinearity variables, due to the chemical similarity (i.e., ORP and DO), high correlation with each other (i.e., Mg^2+^, Ca^2+^ and Sr, Na^+^+K^+^ and SO_4_^2−^), and combination of multiple variables (i.e., TDS), 15 hydrochemical components remained to RDA and heatmap analysis. The RDA results showed that the top two axes accounted for 80% of total cumulative variance, and environmental factors had different proportions that are responsible for the variance on water samples microbial community (Figure 6a and Supplemental Table S4). COD (r^2^ = 0.44, p < 0.05) and NH_4_^+^ (r^2^ = 0.39, *p* < 0.05) were significantly correlated with microbial communities. In particular, COD was positively correlated with the microbial communities of surface waters, while NH_4_^+^ (negatively correlated with ORP) was positively correlated with the microbial communities of goafs, rock roadways with lower ORP, and Ordovician limestone groundwater. The goaf points (i.e., XJL16: closure in 2021; XJL23: closure in 2010; XJL24: closure in 2012; XJL25: closure in 2009) were the most scattered and were strongly correlated with NH_4_^+^, NO_3_^−^, SO_4_^2−^, Fe^2+^, and Fe^3+^, suggesting the microbial community structures of the goafs closure in different times were quite different. The rest points of water sumps, rock roadways, coal roadways, and groundwater aquifers were relatively clustered, mainly distributed in the first quadrant, and positively correlated with SO_4_^2−^, NO_3_^−^, and ORP. Hence, the microbial communities mainly corresponded to the levels of these redox sensitive indices of the water samples in coal mines.

In addition, as shown in the heatmap (Figure 6b), pH, NO_2_^−^, COD, ORP, Fe^3+^, and Fe^2+^ were significantly correlated with the relative abundance of certain microbial communities (e.g., *f_Rhodocyclaceae*, *c_Thermodesulfovibrionia*, *Cavicella*, *Dietzia*, *Luteolibacter*, *f_Rhizobiales_Incertae_Sedis,* etc.) (*p* < 0.05). It was interesting that the correlation of the most top 50 microbial species with NO_2_^−^ and COD was opposite to that of Fe^3+^ and Fe^2+^, possibly due to the coupling relationship between the reducing compounds cycle (e.g., organics and nitrogen) and iron transformation. *Thioclava*, which could accelerate the oxidation of pyrite, was significantly positively correlated with ORP, NO_3_^−^, and SO_4_^2−^, while *c_Thermodesulfovibrionia*, which could reduce SO_4_^2−^, was significantly negatively correlated with pH and COD (*p* < 0.05). *Dietzia* showed a significant positive correlation with SO_4_^2−^, but a significant negative correlation with COD and NO_2_^−^ (*p* < 0.05). *f_Hydrogenophilaceae* was significantly negatively correlated with pH and COD and positively correlated with SO_4_^2^^−^ (*p* < 0.05), which was consistent with pyrite oxidation, followed by increases of H^+^ and SO_4_^2^^−^.

The biotic interactions (e.g., synergistic, antagonistic, and neutral relationships) may also play an important role in shaping the microbial communities [13] (Gao et al., 2019). Co-occurrence networks were constructed of the coal mining water samples to visualize the microbe-microbe interactions (Figure 6c). All the nodes were grouped into five modules with different colors, and most of these correlations (88.71%) were positive. The interactions in each module may be due to the shared niches or a high level of phylogenetic relatedness [34]. Clearly, the nodes corresponding to *Pseudomonas*, *Hydrogenophaga*, and *Thioclava* were identified as the biotic interaction hubs for module 1 (Figure 6c). As shown in Supplemental Figure S7, *Pseudomonas* and *Hydrogenophaga* could use NO_3_^−^ as an alternate electron acceptor and degrade hydrocarbon (e.g., BTEX and naphthalene) [35], and *Pseudomonas* also belonged to Fe(III)-reducing bacteria [13]. *Thioclava* was facultatively anaerobic, with the ability to oxidize S to SO_4_^2−^. Anyway, *Gemmobacter*, *Rhodobacter*, and *Novosphingobium* were regarded as the biotic interaction hubs for module 3 (Figure 6c). *Gemmobacter* could participate in the oxidation of CH_4_ to CO_2_ and dissimilatory NO_3_^−^ reduction [36]. *Rhodobacter* could also reduce NO_3_^−^ and NO_2_^−^ under anaerobic conditions [37], and *Novosphingobium* was capable of degrading hydrocarbon [31]. Notably, module 4 included three biotic interaction hubs (i.e., *f_Rhodocyclaceae*, *o_Burkholderiales*, and *c_Thermodesulfovibrionia*). Among them, *o_Burkholderiales* could degrade organic matter, reduce Fe(III), and participate in the N cycle [38]. *f_Rhodocyclaceae* and *c_Thermodesulfovibrionia* were mainly involved in hydrocarbon degradation and SO_4_^2−^ reduction under anoxic conditions, respectively. In contrast, the most densely connected node was not successfully identified in module 2, and several taxa were visualized in similarly dense connections, such as *Pedomicrobium*, *Dinghuibacter*, *o_Saccharimonadales*, *CL500-29_marine_group*, and *Flavobacterium*. Most of these bacteria were aerobic and mainly involved in the oxidation and accumulation of Fe and Mn, the organic matter fermentation, and the N cycle [39,40]. As a result, these microbial genera, related to the S, N, C, and Fe cycles, had significant correlation with each other and might drive the formation and evolution of hydrochemical characteristics.

### 3.4. Microbial Community Variation Mechanism across Four Typical Environmental Processes in Coal Mine

#### 3.4.1. Vertical Distribution Characteristics of Microbial Communities in Groundwater Aquifers

The main aquifers that affect coal mining in Xinjulong coal mine included S_3_ sandstone, L_3_ limestone, and Ordovician limestone groundwater aquifers, and their vertical distribution structure is shown in Supplemental Figure S8. The hydrochemical compositions and microbial communities of these aquifers showed obvious differences (Supplemental Figure S8a). With the increase of the aquifer buried depth, the pH value gradually became more alkaline (7.9–9.7); the water temperature gradually increased (43–54 °C); the redox conditions evolved to the reducing environment (ORP from 152.5 to −21.9 mV), and NH_4_^+^ gradually increased, while NO_3_^−^ and NO_2_^−^ gradually decreased; SO_4_^2−^ decreased significantly (3.6–0.3 g/L). When sampling in the Ordovician limestone aquifer borehole, an odor of rotten eggs was commonly smelled, indicating the emission of H_2_S gas. The microbial community distribution characteristic corresponded to the hydrochemical results. The results showed that, with the increase of the aquifer buried depth, the microbial community richness and diversity gradually decreased (Supplemental Table S2), and the microbial community gradually changed into an anaerobic and heat-resistant community. The anaerobic and heat-resistant *Candidatus_desulforudis*, which could reduce SO_4_^2−^ to H_2_S, was only detected in the deep Ordovician limestone aquifer (10.28%). However, *Sphingobium*, *Novosphingobium*, *Hyphomonas*, and *Gordonia*, which could degrade organic pollutants, were only detected in the S_3_ sandstone aquifer (1.67–14.33%). Additionally, the FAPROTAX prediction indicated that the functions of aromatic compound degradation and dark oxidation of sulfur compounds were the highest in the S_3_ sandstone groundwater (Supplemental Figure S8b). Because the S_3_ sandstone aquifer occurred on the roof and floor of the 3# coal, its microbial community might be affected by the organic matter and sulfur compounds of the coal seam. These findings may provide support for quickly identifying the source of water inrush (gushing) in the mine.

#### 3.4.2. Variation Characteristics of Microbial Communities during Coal Mining

**Figure 7:**
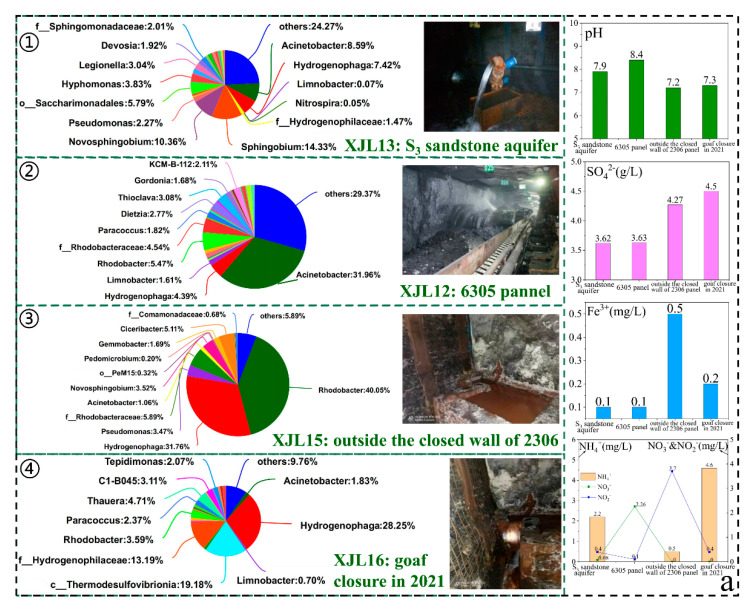

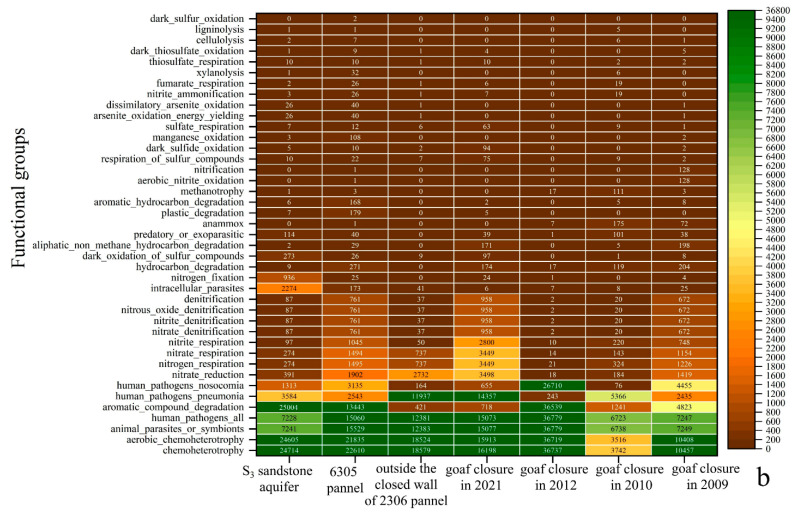
Variation characteristics of microbial community and hydrochemical composition during coal mining (**a**); FAPROTAX function prediction result of coal roadways and goafs (**b**).

Coal mining is mainly carried out on the tunneling panel of the coal roadway. During the tunneling process, S_3_ sandstone groundwater would flow out from the roof and floor of the coal roadway, resulting in a new water–coal/rock reaction. After the panel mining was finished, a closed wall was built to close the goaf. Therefore, we selected ① S_3_ sandstone groundwater, ② 6305 panel, ③ outside the closed wall of 2306 panel, and ④ goaf closure in 2021 (newly closure) as the representative pathway to research the variation rule of microbial community in mine water during coal mining. As shown in Figure 7a, the hydrochemical compositions and microbial communities changed significantly during coal mining. With the advancement of coal mining, the pH value first increased and then decreased, with the highest in the 6305 panel (8.4) and the lowest in the goaf closure in 2021 (7.1); SO_4_^2−^ gradually increased (3.6–4.5 g/L); Fe^2+^ increased first and then decreased; NH_4_^+^ decreased first and then increased, while NO_3_^−^ and NO_2_^−^ did the opposite.

The microbial community richness and diversity of the four sites followed the order of 6305 panel > S_3_ sandstone groundwater > goaf closure in 2021 > outside the closed wall of 2306 panel (Supplemental Table S2). Compared with S_3_ sandstone groundwater, the relative abundance of *Thioclava* that could oxidize low-valence-reducing sulfides into SO_4_^2−^, *Acinetobacter*, *Dietzia*, *Paracoccus*, and *Limnobacter*, which could degrade PAHs and other organic compounds, increased significantly in 6305 panel. The FAPROTAX prediction also showed that the function of dark sulfide oxidation, dark thiosulfate oxidation, manganese oxidation, hydrocarbon degradation, plastic degradation, and aromatic hydrocarbon degradation were the highest in the 6305 panel (Figure 7b). These results confirmed that, on the tunneling panel, pyrite could be oxidized by sulfur-oxidizing bacteria, leading to the increase of SO_4_^2^^−^ concentration, and the organic matter in coal provided abundant carbon source for microorganisms. Meanwhile, higher functions of human pathogens in coal roadways and goafs (Figure 7b) may have resulted from more human mining activities in these zones.

With the goaf closure, the relative abundance of *Rhodobacter*, *f_ Rhodobacteraceae*, and *Gemmobacter* that could reduce NO_3_^−^ or NO_2_^−^ increased significantly outside the closed wall of the 2306 panel (1.7–40.05%), which was consistent with the change trend of NO_3_^−^ or NO_2_^−^ concentration. In addition, in the new closure goaf, groundwater recharge resulted in a short time of more sufficient pyrite oxidation reactions, with higher SO_4_^2−^ and lower pH. Interestingly, the relative abundance of sulfate-reducing bacteria (SRB) increased significantly in the goaf, including *c_Thermodesulfovibrionia* (19.18%), *Desulfobacterium_catecholicum group* (2.3%), *Desulfomicrobium* (2.04%), *f_Desulfuromonadaceae* (1.42%), and *Desulfofustis* (0.82%).

#### 3.4.3. Variation Characteristics of Microbial Communities in Goafs with the Time since Cessation of Mining

**Figure 8:**
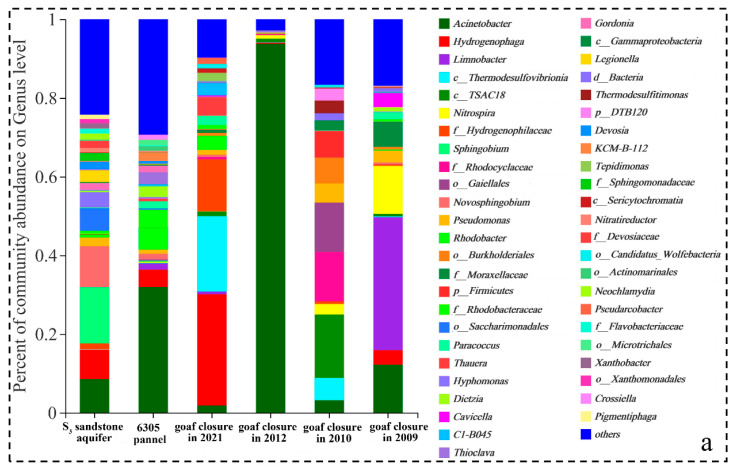

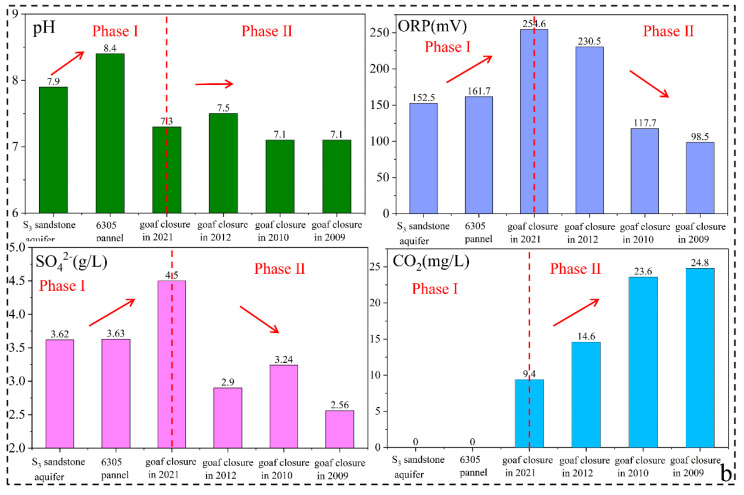
Variation characteristics of microbial community (**a**) and hydrochemical composition (**b**) in goafs with the time, since cessation of mining. Note: Phase I represents coal mining process, and Phase II represents the process since cessation of mining.

Goafs are critical areas of groundwater pollution prevention and control in abandoned coal mines. With the time since goaf closure, the oxygen condition and the degree of water rock interaction change, possibly resulting in the change of the microbial communities and hydrochemical characteristics. Therefore, the goafs that closed in 2021, 2012, 2010, and 2009, whose water-filling aquifers were all S_3_ sandstone groundwater, were selected to study the variation characteristics of microbial communities. We had to make an experimental design of substituting space for time, which may affect the accuracy of the results analysis, but the trends should be similar. As shown in Figure 8b, with the time since goaf closure, the SO_4_^2−^ (4.5–2.6 g/L) and TDS (7.6–4.4 g/L) concentration decreased by 42%, indicating that the long-term closed goafs had a certain self-purification ability. Similarly, Donovan et al. (2003) [41] reported that the SO_4_^2−^ and Fe concentrations of water pumped from an underground acidic coal mine decreased significantly for 4 to 18 years after flooding.

In addition, compared with other zones (i.e., rock roadways, coal roadways, water sumps, and surface waters), the bacteria with sulfate reduction function (i.e., *c_Thermodesulfovibrionia*) and sulfite reduction function (i.e., *Thermodesulfitimonas*) in goafs have relatively higher proportions (Figure 8a). The FAPROTAX prediction also suggested that the function of respiration of sulfur compounds, sulfate respiration, and anammox were higher in goafs (Figure 7b). Therefore, with the time since the cessation of mining, the goafs environment was gradually closed, oxygen was gradually consumed (Supplemental Table S1), and then the desulfurication of SRB occurred in a long-term high SO_4_^2−^ environment. *Acinetobacter*, *o_Burkholderiales*, *Limnobacter*, *Cavicella*, *Paracoccus*, and *Dietzia*, which could degrade hydrocarbon and may generate CO_2_, were detected in different goafs, leading to the increase of free CO_2_ concentration (9.4–24.8 mg/L). It is worth noting that, with the time since goaf closure, more unclassified and rarely reported bacteria were detected. These bacteria may adapt to the environment of oligotrophic and higher heavy metals. The long-term evolution characteristics of hydrochemistry and microbial communities in goaf need to be studied through longer in-situ monitoring and laboratory simulation experiments, which would help predict the potential for natural attenuation and build underground reservoirs in goafs.

#### 3.4.4. Effect of Mine Drainage on Microbial Community of the River

**Figure 9:**
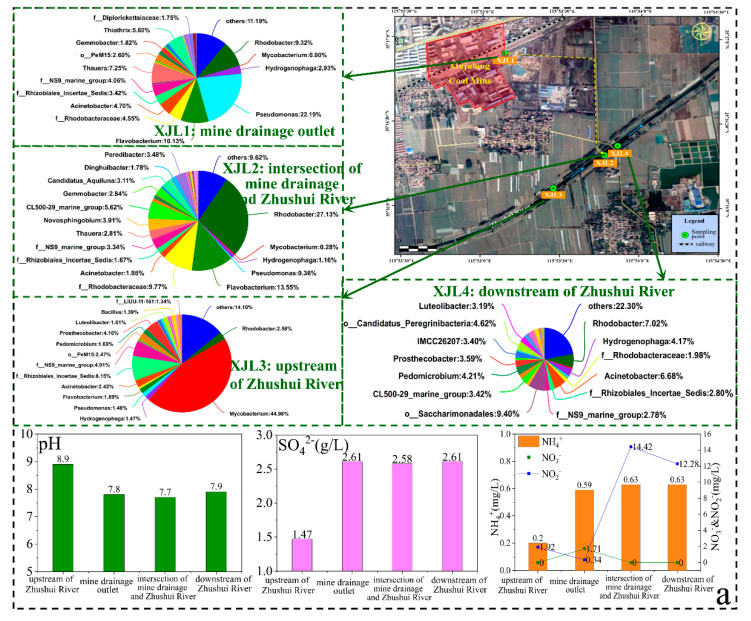

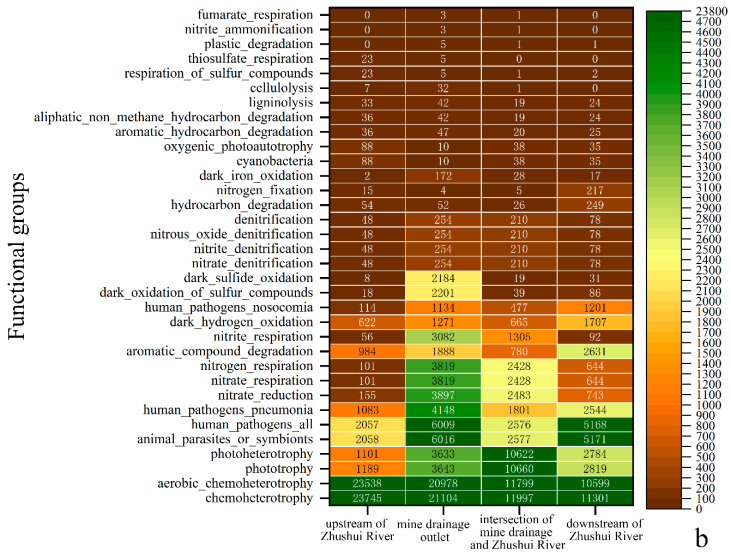
Effects of mine drainage on microbial community and hydrochemical composition in the surface river (**a**); FAPROTAX function prediction result of surface waters (**b**).

In order to ensure safe production from the Xinjulong coal mine, the mine water was discharged into the nearby Zhushui River after treatment both underground and on the ground. The mine drainage outlet and Zhushui River were sampled to study the environmental effect of mine drainage on surface rivers (Figure 9). The conventional hydrochemical compositions concentration (i.e., TDS, K^+^+Na^+^, Ca^2+^, SO_4_^2−^, and Sr) of the mine drainage outlet (XJL1), the intersection of mine drainage and Zhushui River (XJL2), and downstream (XJL4) of Zhushui River were similar, which were significantly higher than those of the river upstream (XJL3). According to the calculation results, based on the ^2^H and ^18^O isotopes, the mine drainage accounted for 59–77% of the downstream flow, with the details described in the Supplementary Materials [42]. From the upstream to the downstream of Zhushui River, DO concentration first decreased and then increased (from 7.35 to 2.62 and then 3.58 mg/L), which was consistent with the larger proportion of aerobic *Mycobacterium* (44.98%) and *f_Rhizobiales_Incertae_Sedis* (8.15%) in XJL3.

What was more noteworthy was that the NO_2_^−^ concentration of XJL1 and XJL3 were both <2 mg/L, but the mine drainage greatly increased the NO_2_^−^ concentration of Zhushui River, with 12.28–14.42 mg/L NO_2_^−^ of XJL2 and XJL4, which was far beyond the quality standard limit of Ⅲ groundwater in China (1 mg/L, GB/T 14848-2017). This phenomenon possibly resulted from the denitrification of *Alsobacter* and *Rhodobacter*, whose relative abundances were significantly higher in XJL2 and XJL4 **(**Figure 9a). The FAPROTAX prediction showed that the functions of denitrification and nitrate reduction were higher in XJL1, XJL2, and XJL4 (Figure 9b). In addition, the upstream temperature was only 16 °C, and the mine drainage temperature was as high as 36.5 °C, resulting in an increase in the downstream temperature (25 °C), which may lead to an increase of alpha diversity and denitrifying bacteria activity in the downstream [43,44]. Therefore, the impact of mine drainage on surface ecological environment is not negligible and needs to be further researched.

## 4. Conclusions

Based on the above findings, the main conclusions are as follows:

(1) The hydrochemical types of mine waters inherited that of their recharge water source (SO_4_-Na type), but their hydrochemical compositions showed obvious zone-specific patterns. The microbial community richness of the six zones was ranked as follows: coal roadways > water sumps ≈ surface waters > rock roadways > goafs > groundwater aquifers, while the microbial community diversity of surface waters was the highest.
(2) The microbial communities in the different zones were eminently different. Bacteria related to sulfur oxidation and hydrocarbon degradation (i.e., *Thioclava* and *Dietzia*) were more abundant in the coal roadways. Anyway, bacteria related to the SO_4_^2−^ reduction, hydrocarbon degradation, and nitrification (i.e., *c_Thermodesulfovibrionia*, *Candidatus_Nitrotoga*, and *Nitrospira*) accounted for higher proportions in goafs and rock roadways. *Sphingobium* and *Candidatus desulforudis* were almost exclusively distributed in groundwater aquifers, while aerobic *Mycobacterium* was the dominant species only in surface waters.
(3) The RDA and heatmap analysis demonstrated that the microbial communities corresponded to the levels of redox sensitive indices (i.e., COD, NH_4_^+^, NO_2_^−^, ORP, SO_4_^2−^, Fe^3+^, and Fe^2+^). Co-occurrence networks, with 88.71% positive correlations, confirmed that these microbial genera, related to the S, N, C, and Fe cycles, had significant correlation with each other and might drive the formation and evolution of hydrochemical characteristics.
(4) Coal roadways and goafs were the critical zones of groundwater pollution prevention and control. During tunneling in the panel, pyrite could be oxidized by sulfur-oxidizing bacteria, leading to SO_4_^2^^−^ increase. With the closure of the panel and the formation of the goaf, the SO_4_^2−^ concentration increased rapidly for a short period. However, with the time since goaf closure, the proportion of SRB increased significantly, leading to SO_4_^2−^ concentration’s decrease by 42% over 12 years, which indicated that long-term closed goafs had a certain self-purification ability.

Altogether, this research provides a new insight for clarifying on the formation and evolution mechanisms of the special mine water quality in different zones and developing groundwater pollution prevention and control technology. More work should explore the significance of microbial communities as bioindicators for monitoring mining activities and their potential uses in bioremediation.

## Data Availability

The raw reads were submitted into the NCBI Sequence Read Archive database (accession number: PRJNA818133).

## Supplementary Materials

The following supporting information can be downloaded at: https://www.mdpi.com/article/10.3390/ijerph192013359/s1, Materials and Methods–sampling points design; Table S1: Sampling place, concentrations of in-situ physicochemical parameters, major and trace chemical constituents of water samples in different zones of Xinjulong coal mine; Table S2: Alpha diversity of microbial community; Table S3: Microbial community structure of different zones water samples on different levels (Appendix I); Table S4: The envfit environment factors of RDA; Table S5: Calculation results of contribution ratio (R) of mine drainage to Zhushui River downstream; Table S6: Comparison table of genus raw classification output and abbreviated names; Figure S1: Representative field photos of sampling sites in Xinjulong coal mine and mine water discharged to Zhushui River in Shandong Province, China; Figure S2: Rarefication curves after flattening; Figure S3: Half-box-and-whisker diagrams of the hydrochemical components in the six zones, except for Figure 4; Figure S4: The numbers of core species, unique species, and shared species of the microorganisms in the six zones of Xinjulong coal mine on genus level (Venn diagram); Figure S5: Beta diversity of microbial community. PCoA plot of microbial communities in water samples (**a**). ANOSIM of microbial communities in six different zones (**b**); Figure S6: Kruskal–Wallis H test of distribution of microbial phyla (**a**) and genera (**b**) in different zones’ water samples (* 0.01 < *p* ≤ 0.05, ** 0.001 < *p* ≤ 0.01); Figure S7: Conceptual model of hydrocarbon degradation by *Pseudomonas* and *Hydrogenophaga*, with NO_3_^−^ as an alternate electron acceptor; Figure S8: Vertical distribution characteristics of microbial community and hydrochemical composition in groundwater aquifers (**a**); FAPROTAX function prediction result of groundwater aquifers (**b**); Figure S9: δ^2^H-δ^18^O plot for water samples. Note: GMWL refers to the global meteoric water line; LMWL refers to the local meteoric water line.
