# Supplementary material for: Coal Mining Activities Driving the Changes in Microbial Community and Hydrochemical Characteristics of Underground Mine Water
Source: Int J Environ Res Public Health. 2022 Oct 16;19(20):13359. doi: 10.3390/ijerph192013359 (PMC9603172; doi:10.3390/ijerph192013359)
Supplement: Supplementary file 1 [file ijerph-19-13359-s001.zip › Supplementary material-clean.pdf]

Coal mining activities driving the changes in microbial community and hydrochemical characteristic of underground mine water

Li Zhang[a] Zhimin Xu[a,b]* Yajun Sun[a,b] Yating Gao[a] Lulu Zhu[a]

a School of Resources and Geosciences, China University of Mining and Technology, Xuzhou, Jiangsu 221116, China.

b Fundamental Research Laboratory for Mine Water Hazards Prevention and Controlling Technology, Xuzhou, Jiangsu 221006, China

Corresponding author:

Zhimin Xu

School of Resources and Geosciences, China University of Mining and Technology, Xuzhou, Jiangsu 221116, China

E-mail: xuzhimin@cumt.edu.cn

Tel. +86-13775978891

Materials and methods–sampling points design

The groundwater from water-filling aquifers was the origination of mine water. Rock roadway referred to the roadway with the rock area higher than 80% in the excavation section, which was mainly used for ventilation, transportation equipment and materials, etc. The 4 rock roadways sampling points passed through the L_3 limestone aquifer. Thus, most of the rock roadways water originated from the L_3 limestone groundwater and collected in the drainage ditches of the rock roadways, except for XJL14 which collected the drainage of coal roadways and rock roadways. Coal roadway referred to the roadway with coal area higher than 80% in the excavation section including pannel, which was mainly used for mining and transporting coal. The main water-filling aquifer of coal roadways was S_3 sandstone aquifer. After cessation of mining, a closed wall would be built to close the pannel; the pannel was called the goaf at this time. Then in goaf, the water level increased gradually and the oxygen concentrations decreased gradually with the time since goaf closure, resulting in a long-term and specific water-coal/rock reaction. Thus, the goafs closure in 2021, 2012, 2010 and 2009, whose water-filling aquifers were all S_3 sandstone groundwater, were selected to study the variation characteristics of microbial communities. Here, we had to make the experimental design of substituting space for time, because the microbial community and hydrochemical characteristic of goafs closure several years ago were not detected. Water sump was used to temporarily store underground mine water and sediment of the whole mine, which collected the water and sediment from groundwater aquifers, rock roadways, coal roadways and goafs, etc. The mine water in the water

sumps was discharged to the surface Zhushui River after treatment. In order to research the effect of mine drainage on microbial community of the river, the mine drainage outlet (XJL1), the intersection of mine drainage and Zhushui River (XJL2), the upstream of Zhushui River (XJL3) and the downstream (XJL4) of Zhushui River were sampled.

Table S1 Sampling place, concentrations of in-situ physicochemical parameters, major and trace chemical constituents of water samples in different zones of Xinjulong Coal Mine.

Group	Sample name	sampling place	pH	DO mg/L	ORP mV	K^++Na^+ mg/L	Ca^{2+} mg/L	Mg^{2+} mg/L	Fe^{3+} mg/L	Fe^{2+} mg/L	NH_4^+ mg/L	Sr mg/L	Cl^- mg/L	SO_4^{2-} mg/L	HCO_3^- mg/L	CO_3^{2-} mg/L	NO_3^- mg/L	NO_2^- mg/L	TDS mg/L	CO_2 mg/L	H_2SiO_3 mg/L	COD mg/L
GW	XJL9	Ordovician limestone aquifer	9.7	2.67	-21.9	492.0	1.6	1.4	0.11	0.09	3.18	0.8	394.2	310.4	138.6	57.5	<LQ	0.02	1356	<LQ	4.1	5.19
	XJL13	S_3 sandstone aquifer	7.9	4.57	152.5	1312.0	265.2	128.2	0.05	<LQ	2.20	10.6	55.9	3618.0	236.5	4.8	0.08	0.42	5772	<LQ	12.1	6.62
	XJL27	L_3 limestone aquifer	7.8	2.25	96.0	1210.4	63.1	39.7	0.16	<LQ	2.05	7.5	211.2	2246.5	392.6	—	<LQ	0.11	4084	6.5	16.0	3.25
RR	XJL14	air inlet in No.1 auxiliary roadway	8.6	4.33	231.2	1378.6	283.7	152.1	0.04	0.05	0.01	6.9	121.1	3767.4	248.7	19.2	2.66	0.02	6098	<LQ	10.8	1.88
	XJL18	No.2 auxiliary roadway drainage ditch	8.4	4.02	177.2	734.2	348.0	104.3	<LQ	<LQ	1.89	10.9	299.1	2231.3	187.8	—	<LQ	0.04	4054	9.4	13.9	1.64
	XJL19	No.1 auxiliary roadway drainage ditch	8.5	3.83	179.8	1044.7	333.5	116.0	0.05	<LQ	<LQ	10.4	236.8	2932.7	190.2	21.6	1.84	0.23	4962	<LQ	12.5	1.61
	XJL26	No.2 auxiliary roadway	7.5	2.71	74.1	1002.5	337.5	112.1	0.13	0.05	0.20	11.2	242.1	2833.5	213.6	9.6	1.25	0.40	4780	<LQ	13.1	1.28
CR	XJL12	6305 pannel	8.4	4.75	161.7	1412.5	261.2	135.5	0.06	<LQ	0.01	6.3	131.7	3626.6	372.1	7.2	2.26	0.05	6016	<LQ	10.6	1.14
	XJL15	outside the closed wall of 2306 pannel	7.2	2.84	171.4	1665.5	339.9	120.4	0.49	<LQ	0.45	8.5	147.1	4268.3	380.4	—	<LQ	3.74	6950	12.9	10.7	3.38
goaf	XJL16	goaf closure in 2021	7.3	2.96	254.6	2013.5	245.1	85.3	0.19	0.19	4.63	5.4	97.9	4500.9	646.1	—	<LQ	0.35	7614	9.4	9.5	1.34

Group	Sample name	sampling place	pH	DO mg/L	ORP mV	K^{+}+Na^{+} mg/L	Ca^{2+} mg/L	Mg^{2+} mg/L	Fe^{3+} mg/L	Fe^{2+} mg/L	NH_4^{+} mg/L	Sr mg/L	Cl^{-} mg/L	SO_4^{2-} mg/L	HCO_3^{-} mg/L	CO_3^{2-} mg/L	NO_3^{-} mg/L	NO_2^{-} mg/L	TDS mg/L	CO_2 mg/L	H_2SiO_3 mg/L	COD mg/L
	XJL23	goaf closure in 2010	7.1	2.86	117.7	1157.7	345.6	157.4	0.07	0.05	2.80	6.3	235.0	3239.3	403.8	—	<LQ	0.06	5462	23.6	14.5	1.67
	XJL24	goaf closure in 2012	7.5	3.22	230.5	1066.4	313.4	129.1	0.08	<LQ	<LQ	9.5	246.8	2900.1	321.9	—	1.12	0.01	4918	14.6	15.4	1.30
	XJL25	goaf closure in 2009	7.1	2.70	98.5	869.7	365.7	117.0	0.09	<LQ	2.03	9.3	258.2	2559.3	319.4	—	<LQ	0.01	4428	24.8	15.4	1.14
sump	XJL17	extend the lower water sump	7.6	3.70	254.0	1546.9	291.7	117.0	0.26	<LQ	0.01	6.9	154.9	3899.9	356.0	—	3.18	0.02	6368	11.1	11.2	1.51
	XJL20	1# water sump	8.2	3.75	150.8	1102.7	277.2	117.9	0.15	<LQ	<LQ	8.3	229.0	2925.7	221.4	13.4	2.46	0.17	4996	<LQ	11.1	1.80
	XJL21	2# water sump	7.7	2.70	66.5	1015.1	196.9	74.1	<LQ	<LQ	1.67	6.9	250.3	2383.6	186.8	12.0	<LQ	0.01	4136	<LQ	11.1	1.52
	XJL22	level 2 water sump	8.1	2.73	135.5	1264.0	221.0	73.1	0.05	0.30	0.11	7.3	264.6	2912.5	239.0	—	<LQ	0.09	4890	4.3	6.5	1.44
SW	XJL1	mine drainage outlet	7.8	5.33	87.4	1014.9	286.8	78.3	0.03	<LQ	0.59	7.3	262.1	2606.3	197.0	—	1.71	0.34	4448	5.3	15.6	3.43
	XJL2	intersection of mine drainage and Zhushui River	7.7	2.62	208.6	986.6	278.0	94.6	0.03	<LQ	0.63	7.2	270.8	2584.0	173.3	—	<LQ	14.42	4402	17.6	11.4	8.24
	XJL3	upstream of Zhushui River	8.9	7.35	150.2	577.4	138.0	122.5	0.05	<LQ	0.20	3.3	259.3	1469.4	252.5	—	<LQ	1.92	2750	7.8	<LQ	7.92
	XJL4	downstream of Zhushui River	7.9	3.58	204.1	1000.7	277.2	92.7	0.05	<LQ	0.63	7.0	265.6	2612.4	174.3	—	<LQ	12.28	4436	12.3	9.9	7.92
Limit of Quantification (LQ)			0.0	0.0	-2000	0.04	0.01	0.05	0.03	0.03	0.01	0.01	10	10	—	—	0.08	0.01	20	0.1	0.1	0.5
Bounds of Absolute Error (precision)			0.02	0.10	1.0	50	2	2	0.1	0.1	0.25	0.5	4	40	6	2	0.15	0.1	20	1.2	0.5	0.2

Note: GW, RR, CR, goaf, sump and SW refer to groundwater aquifers, rock roadways, coal roadways, goafs, water sumps and surface waters, respectively. DO, ORP, TDS and COD refer to dissolved oxygen, oxidation-reduction potential, total dissolved solids and chemical oxygen demand, respectively.

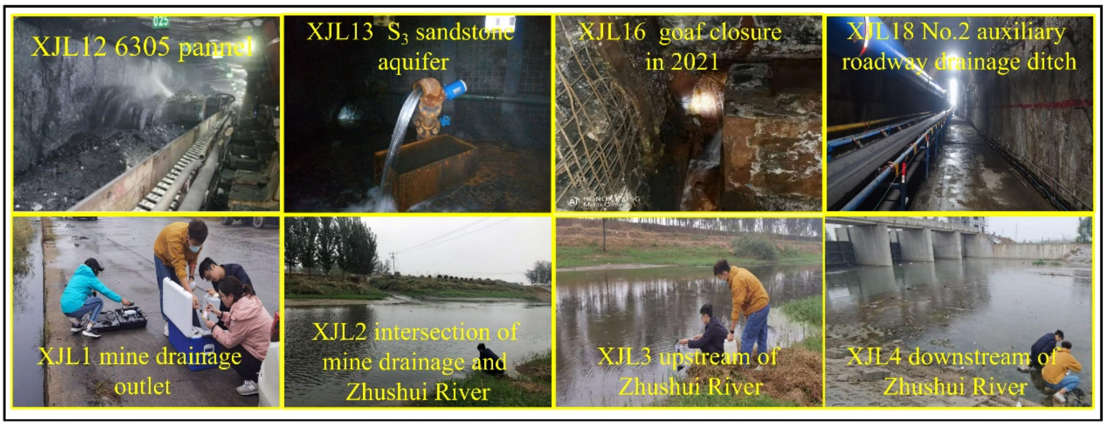

Figure S1. Representative field photos of sampling sites in Xinjulong Coal Mine and mine-water-discharged Zhushui River in Shandong Province, China.

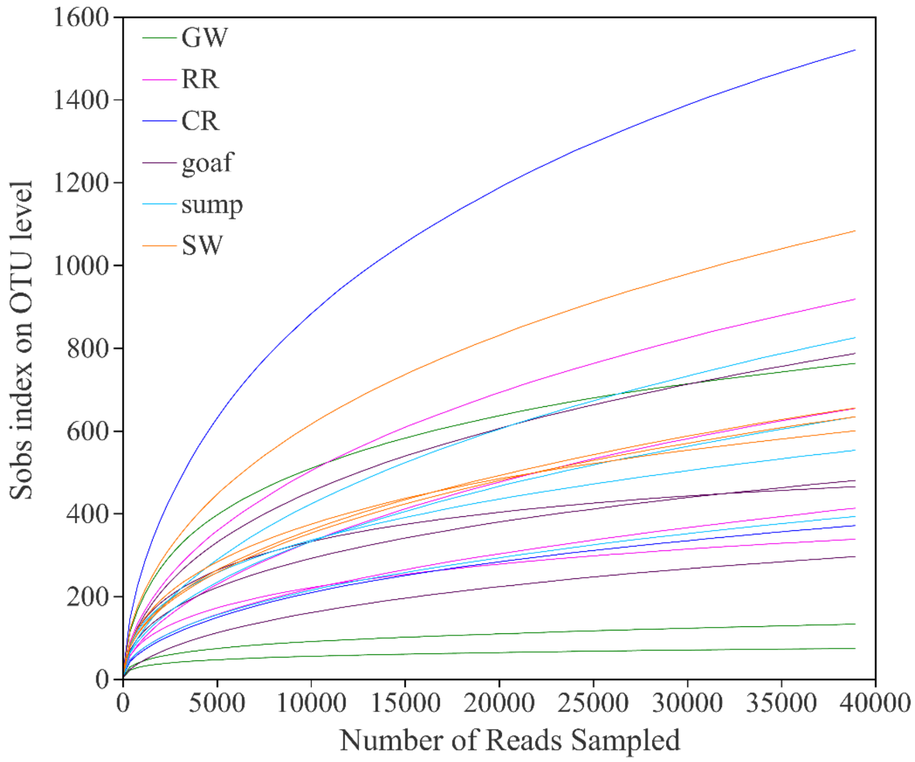

Figure S2. Rarefication curves after flattening.

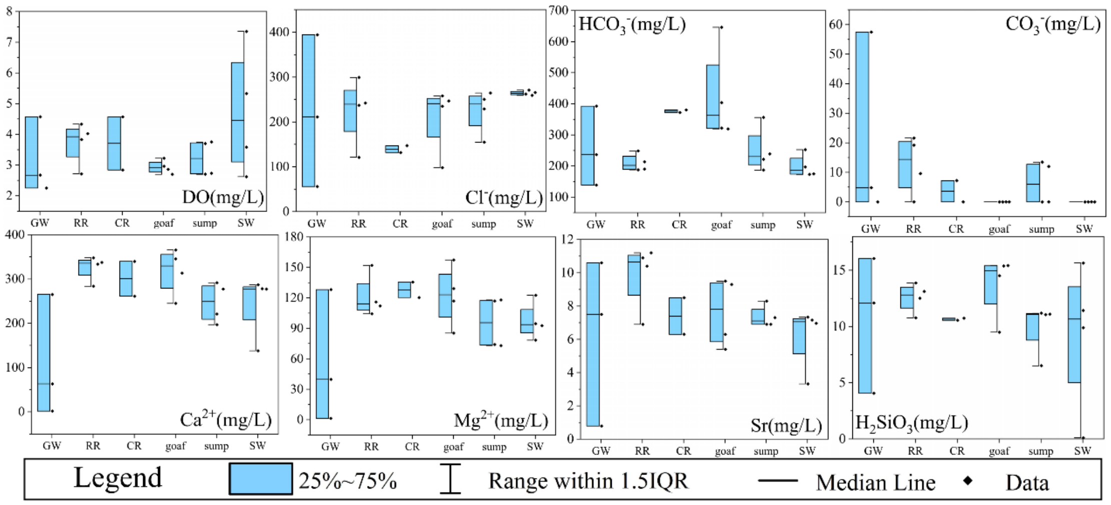

Figure S3. Half-box and whisker diagrams of the hydrochemical components in the six zones except for **Figure 4**. Note: GW, RR, CR, goaf, sump and SW refer to groundwater aquifers, rock roadways, coal roadways, goafs, water sumps and surface waters, respectively.

$$2FeS_2+7O_2+2H_2O \rightarrow 2FeSO_4+2H_2SO_4 \quad (S1)$$

$$2FeSO_4+H_2SO_4+1/2O_2 \rightarrow Fe_2(SO_4)_3+H_2O \quad (S2)$$

$$Fe_2(SO_4)_3+2H_2O \rightarrow 2Fe(OH)SO_4+H_2SO_4 \quad (S3)$$

Table S2 Alpha diversity of microbial community.

Group	Sample name	Sequence	OTUs	Coverage (%)	Richness		Diversity	
					ACE	Chao1	Shannon	Simpson
GW	XJL9	61841	73	99.96%	88.12	88	1.62	0.34
	XJL13	61206	762	99.51%	944.29	933	4.31	0.04
	XJL27	49812	132	99.89%	218.8	200.33	1.91	0.31
RR	XJL14	64092	412	99.50%	997.29	768.31	2.74	0.14
	XJL18	60940	917	99.05%	1693.65	1375.76	3.67	0.08
	XJL19	54909	653	99.25%	1358.83	1012.48	3.27	0.07
	XJL26	52183	337	99.76%	430.93	438.86	2.84	0.16
CR	XJL12	67922	1519	98.67%	2084.25	2100.91	4.71	0.04
	XJL15	54598	370	99.63%	659.32	545.05	2.48	0.19
goaf	XJL16	66979	479	99.59%	777.64	655.92	3.4	0.08
	XJL23	41385	664	99.79%	519.63	514.63	3.7	0.06
	XJL24	54314	295	99.69%	534.65	463.84	1.22	0.5
	XJL25	49446	786	99.23%	1434.64	1205.16	3.21	0.15
sump	XJL17	70923	392	99.57%	814.49	624.12	2.98	0.09
	XJL20	62955	552	99.49%	979.89	867.87	3.55	0.08
	XJL21	57794	824	99.07%	1661.9	1292.55	3.37	0.08

Group	Sample name	Sequence	OTUs	Coverage (%)	Richness		Diversity	
					ACE	Chao1	Shannon	Simpson
	XJL22	59291	633	99.26%	1387.76	1060.51	3.02	0.14
SW	XJL1	74250	633	99.35%	1178.71	943.06	3.69	0.06
	XJL2	73472	654	99.30%	1356.11	1040.75	3.69	0.06
	XJL3	57360	599	99.53%	795.43	806.56	3.39	0.11
	XJL4	63845	1082	98.95%	1604.21	1656.28	4.68	0.02

Note: GW, RR, CR, goaf, sump and SW refer to groundwater aquifers, rock roadways, coal roadways, goafs, water sumps and surface waters, respectively.

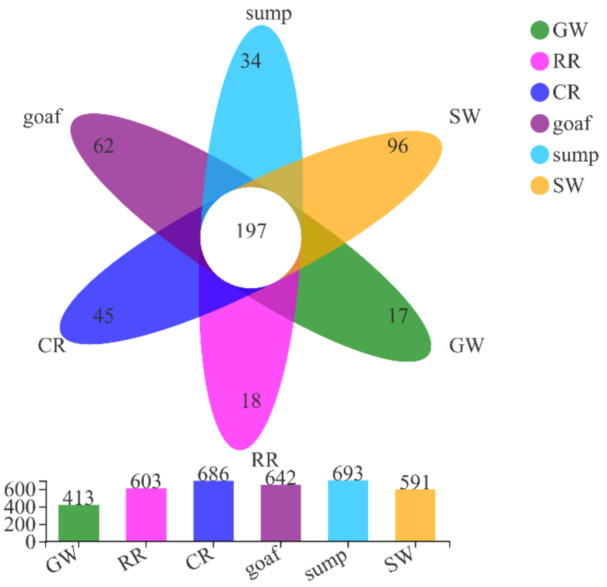

Figure S4. The numbers of core species, unique species and shared species of the microorganisms in the six zones of Xinjulong Coal Mine on genus level (Venn diagram). Note: GW, RR, CR, goaf, sump and SW refer to groundwater aquifers, rock roadways, coal roadways, goafs, water sumps and surface waters, respectively.

Beta diversity analysis (e.g., PCoA and ANOSIM) is commonly used to compare microbial community compositions through quantifying similarity and dissimilarity of microbial samples. According to the result of PCoA, two main factors that made the biggest contribution for differences between samples which were accounted for 32.66% and 19.84%, respectively (**Figure S5a**). ANOSIM further confirmed that six zones of water samples had significant differences (R^2=0.30, p<0.05, **Figure S5b**). Compared

with other zones, the intragroup variations of microbial community structures in groundwater aquifers and coal roadways were higher, while those in surface waters and water sumps were lower.

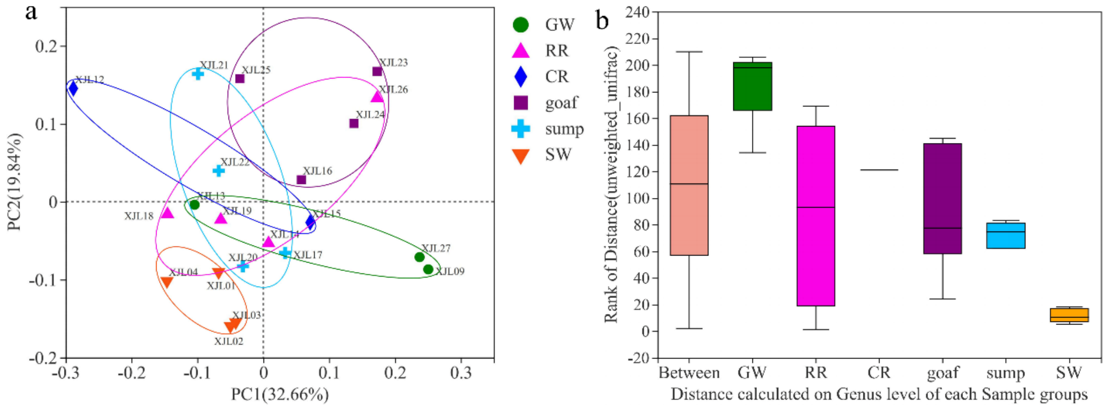

Figure S5. Beta diversity of microbial community. PCoA plot of microbial communities in water samples (a). ANOSIM of microbial communities in six different zones (b). Note: GW, RR, CR, goaf, sump and SW refer to groundwater aquifers, rock roadways, coal roadways, goafs, water sumps and surface waters, respectively.

Table S3. Microbial community structure of different zones water samples on different levels (Appendix I).

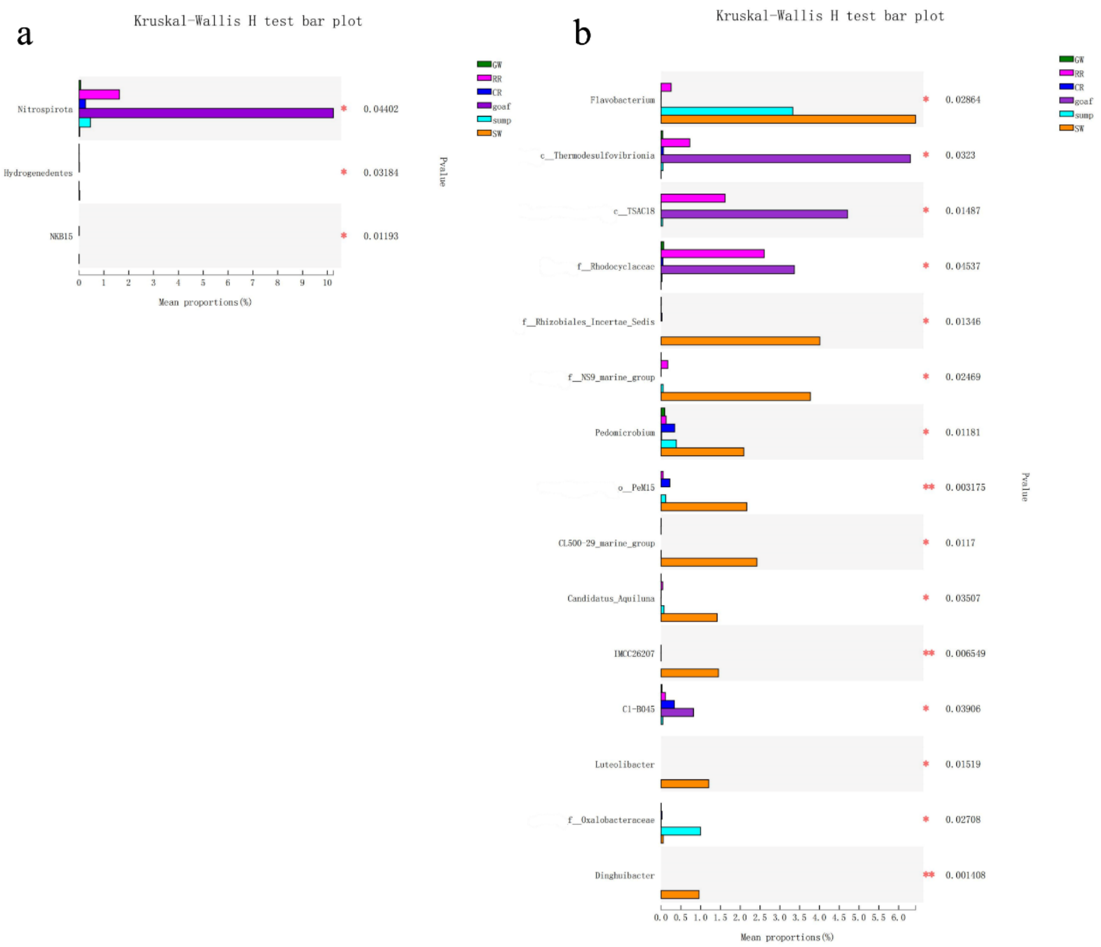

Figure S6. Kruskal-Wallis H test of distribution of microbial phyla (a) and genera (b) in different zons water samples. (*0.01<p≤0.05, ** 0.001<p≤0.01). Note: GW, RR, CR, goaf, sump and SW refer to groundwater aquifers, rock roadways, coal roadways, goafs, water sumps and surface waters, respectively.

Table S4. The envfit environment factors of RDA.

Environmental variables	RDA1	RDA2	r^2	p values
pH	-0.750	0.661	0.021	0.818
ORP	0.186	0.983	0.189	0.17
Ca	0.810	0.586	0.016	0.859
Fe^{3+}	0.934	-0.357	0.077	0.39
Fe^{2+}	0.382	-0.924	0.109	0.245
NH_4^+	-0.104	-0.995	0.388	0.019
Cl^-	-0.655	-0.756	0.094	0.412
SO_4^{2-}	0.949	0.316	0.101	0.383
HCO_3^-	0.326	-0.945	0.039	0.677
CO_3^{2-}	0.302	-0.953	0.236	0.132
NO_3^-	0.862	0.507	0.120	0.32
NO_2^-	-0.831	0.557	0.213	0.149

Environmental variables	RDA1	RDA2	r^2	*p* values
CO_2	-0.874	-0.486	0.108	0.326
H_2SiO_3	0.943	-0.332	0.209	0.127
COD	-0.886	0.463	0.440	0.014

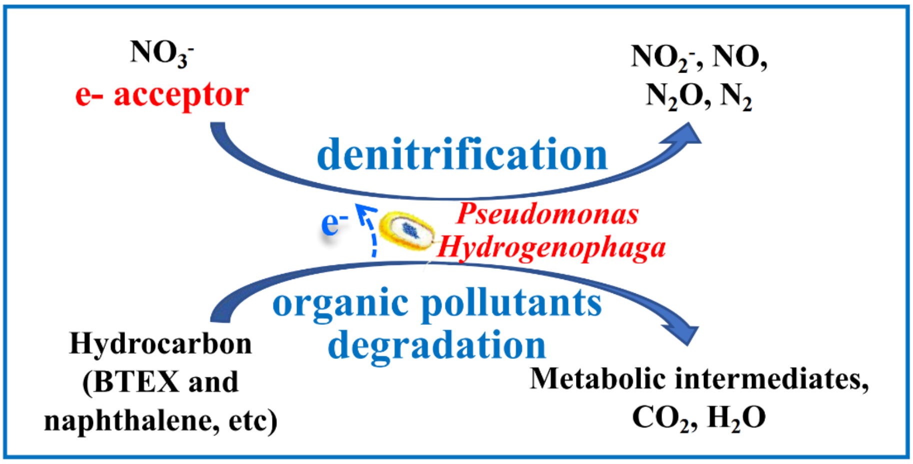

Figure S7. Conceptual model of hydrocarbon degradation by *Pseudomonas* and *Hydrogenophaga* with NO_3^- as an alternate electron acceptor.

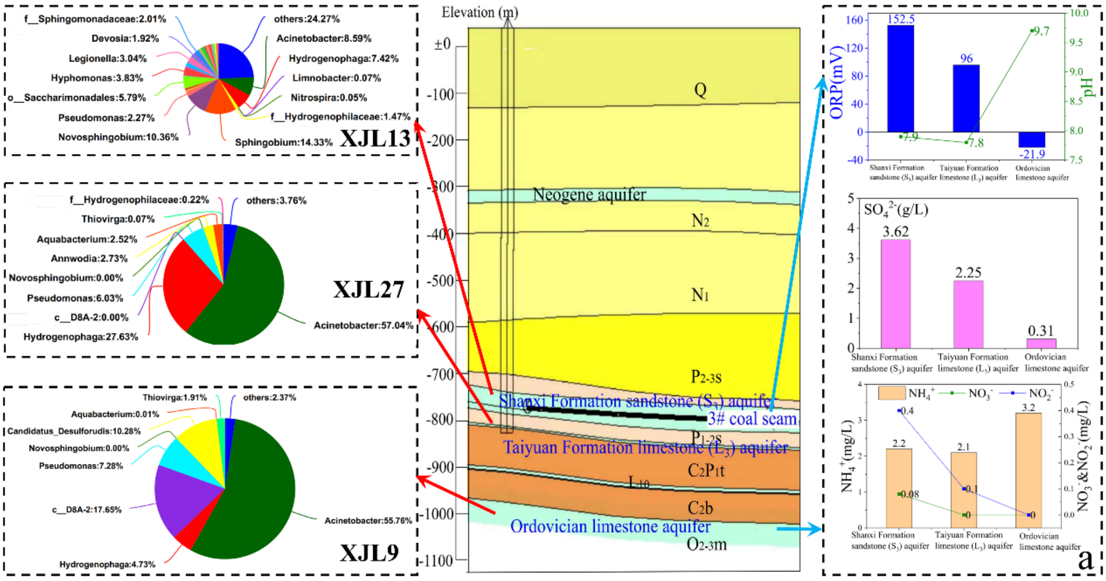

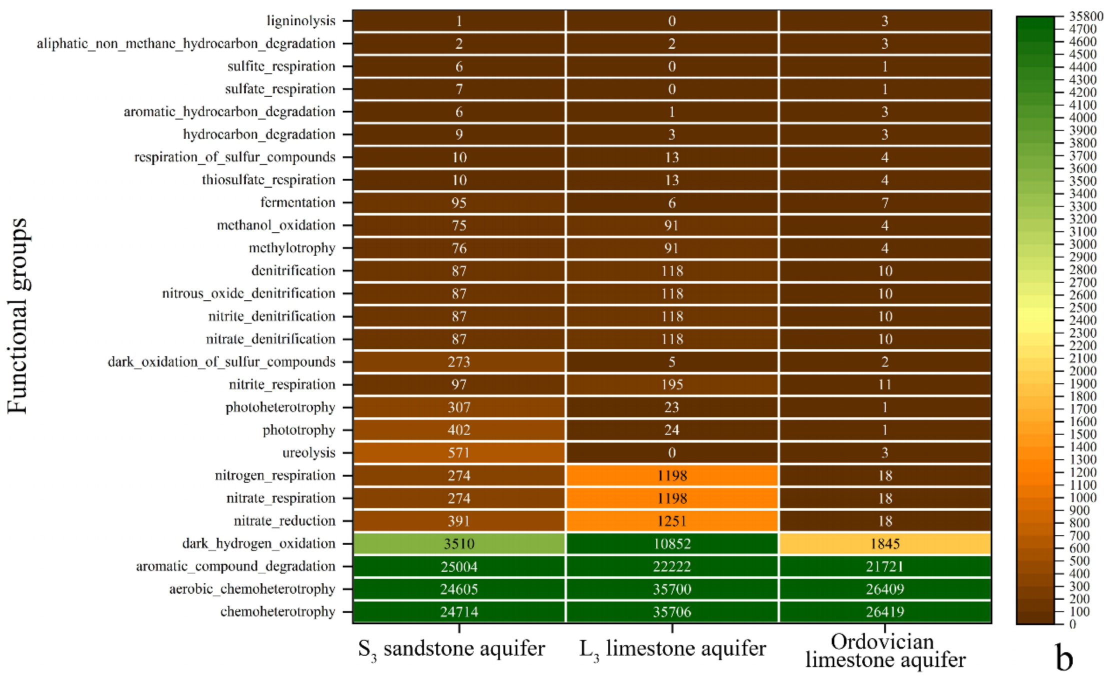

Figure S8. Vertical distribution characteristics of microbial community and hydrochemical composition in groundwater aquifers (a); FAPROTAX function prediction result of groundwater aquifers (b).

The contribution calculation of mine drainage to Zhushui River

The waters from the mine drainage outlet (XJL1) and the upstream of Zhushui River (XJL3) were mixed and flowed into the downstream of Zhushui River (XJL4). To calculate the contribution of mine drainage to Zhushui River, δ^2H and $\delta^{18}O$ of these surface water samples were detected by gas stable isotope mass spectrometer (MAT253-EA, Thermo Fisher Scientific Co. LTD., USA). The detection results are presented in thousandth difference (‰) relative to Vienna Standard Mean Ocean Water (VSMOW), with the precision of ±2.0‰ for δ^2H and ±0.1‰ for $\delta^{18}O$. As shown in **Table S5** and **Figure S9**, the ^2H and ^{18}O abundance fractional isotope of XJL1 was obviously different with that of XJL3. The distances between the three sampling points were less than 1 km, so isotopic fractionation was negligible due to evaporation and water-rock interaction. On this basis, the isotope method (Eq. S4–S5) could be used to

calculate the contribution ratios of mine drainage and the upstream water to the downstream mixed water of Zhushui River (Qian and Ma, 2005). XJL1 and XJL3 were regarded as end-members samples, and XJL4 was regarded as a mixed sample.

$$\delta_1 \times R + \delta_3 \times (1 - R) = \delta_4 \quad \text{(S4)}$$

$$R = \frac{\delta_4 - \delta_3}{\delta_1 - \delta_3} \quad \text{(S5)}$$

where R is the contribution ratio of mine drainage to the downstream mixed water of Zhushui River; $(1-R)$ is the contribution ratio of the upstream water to the downstream mixed water of Zhushui River; δ_1, δ_3 and δ_4 (‰) are δ^2H or δ^{18}O of XJL1, XJL3 and XJL4, respectively.

The calculated value of R is shown in **Table S5**, which indicated that the mine drainage accounted for 59–77% of the downstream flow.

Table S5. Calculation results of contribution ratio (R) of mine drainage to Zhushui River downstream.

Sample name	δ^2H (‰)	R_1	δ^{18}O (‰)	R_2
XJL1 mine drainage outlet	-65.4		-9.35	
XJL3 upstream of Zhushui River	-54.1	76.99%	-6.91	59.43%
XJL4 downstream of Zhushui River	-62.8		-8.36	

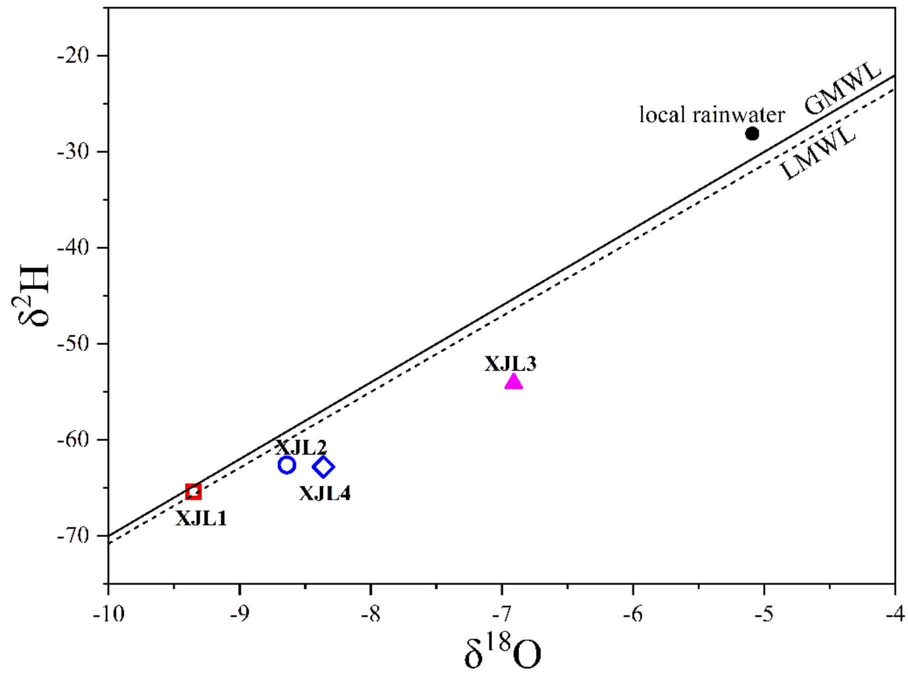

Figure S9. δ^2H-δ^{18}O plot for water samples. Note: GMWL refers to the global meteoric water line; LMWL refers to the local meteoric water line.

Table S6. Comparison table of genus raw classification output and abbreviated names.

Raw classification output names	Abbreviated names
norank_f__norank_o__norank_c__Thermodesulfovibrionia	*c_Thermodesulfovibrionia*
norank_f__Rhodocyclaceae	*f_Rhodocyclaceae*
norank_f__Rhizobiales_Incertae_Sedis	*f_Rhizobiales_Incertae_Sedis*
norank_f__Hydrogenophilaceae	*f_Hydrogenophilaceae*
unclassified_o__Burkholderiales	*o_Burkholderiales*
norank_f__norank_o__Saccharimonadales	*o_Saccharimonadales*
unclassified_f_Rhodobacteraceae	*f_Rhodobacteraceae*
norank_f__Desulfuromonadaceae	*f_Desulfuromonadaceae*
Norank_f_Rhizobiales_Incertae_Sedis	*f_Rhizobiales_Incertae_Sedis*

Note: Species annotated with "no rank" are those for which the sequences can be matched against the Silva v138 16S rRNA database at a taxonomic level, but no specific taxonomic information is available. In this case, a higher level of taxonomic information will be annotated in the statistics. In addition, "c", "o", and "f" represent Class, Order, and Family, respectively. For example, *norank_f__Rhodocyclaceae* is identified and named on genus level according to the database; the family level information of this genus has been determined, but the genus level has not been determined. Generally, *norank_f__Rhodocyclaceae* is considered to have a similar function to *Rhodocyclaceae*. Moreover, species annotated with "unclassified" are those for which the sequences

cannot be matched against the Silva v138 16S rRNA database with a confidence threshold of 0.7 at a taxonomic level.

REFERENCES

Qian, H.; Ma, Z.Y. *Hydrogeochemistry*, 1st ed.; Geology Press: Beijing, China, 2005; pp. 134.
